# Hepatic myofibroblasts exert immunosuppressive effects independent of the immune checkpoint regulator PD-L1 in liver metastasis of pancreatic ductal adenocarcinoma

**DOI:** 10.3389/fonc.2023.1160824

**Published:** 2023-05-03

**Authors:** Silje Beckinger, Tina Daunke, Leon Aldag, Sandra Krüger, Steffen Heckl, Daniela Wesch, Heiner Schäfer, Christoph Röcken, Sascha Rahn, Susanne Sebens

**Affiliations:** ^1^Institute for Experimental Cancer Research, Kiel University and University Hospital Schleswig-Holstein Campus Kiel, Kiel, Germany; ^2^Department of Pathology, University Hospital Schleswig-Holstein Campus Kiel, Kiel, Germany; ^3^Department of Internal Medicine II, University Hospital Schleswig-Holstein Campus Kiel, Kiel, Germany; ^4^Institute of Immunology, Kiel University and University Hospital Schleswig-Holstein Campus Kiel, Kiel, Germany; ^5^Institute of Biochemistry, Kiel University, Kiel, Germany

**Keywords:** pancreatic cancer, immune evasion, 3D coculture, hepatic metastasis, programmed death ligand 1, tumor microenvironment

## Abstract

**Introduction:**

Pancreatic ductal adenocarcinoma (PDAC) represents the 4^th^ most common cause of cancer-related deaths in Western countries. Most patients are diagnosed at advanced stages, often already with metastases. The main site of metastasis is the liver and hepatic myofibroblasts (HMF) play a pivotal role in metastatic outgrowth. Immune checkpoint inhibitors (ICI) targeting programmed death ligand 1 (PD-L1) or programmed cell death protein 1 (PD-1) improved treatment of several cancers but not of PDAC. Therefore, this study aimed to better understand the impact of HMF on PD-L1 expression and immune evasion of PDAC cells during liver metastasis.

**Methods:**

Formalin-fixed and paraffin embedded biopsy samples or diagnostic resection specimens from liver metastases of 15 PDAC patients were used for immunohistochemical analyses. Serial sections were stained with antibodies directed against Pan-Cytokeratin, αSMA, CD8, and PD-L1. To investigate whether the PD-1/PD-L1 axis and HMF contribute to immune escape of PDAC liver metastases, a stroma enriched 3D spheroid coculture model was established *in vitro*, using two different PDAC cell lines, HMF, and CD8^+^ T cells. Here, functional and flow cytometry analyses were conducted.

**Results:**

Immunohistochemical analysis of liver tissue sections of PDAC patients revealed that HMF represent an abundant stroma population in liver metastases, with clear differences in the spatial distribution in small (1500 µm) and large (> 1500 μm) metastases. In the latter, PD-L1 expression was mainly located at the invasion front or evenly distributed, while small metastases either lacked PD-L1 expression or showed mostly weak expression in the center. Double stainings revealed that PD-L1 is predominantly expressed by stromal cells, especially HMF. Small liver metastases with no or low PD-L1 expression comprised more CD8^+^ T cells in the tumor center, while large metastases exhibiting stronger PD-L1 expression comprised less CD8^+^ T cells being mostly located at the invasion front. HMF-enriched spheroid cocultures with different ratios of PDAC cells and HMF well mimicking conditions of hepatic metastases *in situ*. Here, HMF impaired the release of effector molecules by CD8^+^ T cells and the induction of PDAC cell death, an effect that was dependent on the amount of HMF but also of PDAC cells. ICI treatment led to elevated secretion of distinct CD8^+^ T cell effector molecules but did not increase PDAC cell death under either spheroid condition.

**Conclusion:**

Our findings indicate a spatial reorganization of HMF, CD8^+^ T cells, and PD-L1 expression during progression of PDAC liver metastases. Furthermore, HMF potently impair the effector phenotype of CD8^+^ T cells but the PD-L1/PD-1 axis apparently plays a minor role in this scenario suggesting that immune evasion of PDAC liver metastases relies on other immunosuppressive mechanisms.

## 1 Introduction

Pancreatic ductal adenocarcinoma (PDAC) is the 4^th^ most common cause of cancer-related deaths in Western countries with a 5-year survival rate of less than 10% (1). Lacking specific symptoms, PDAC is lately (80%) diagnosed at a locally advanced or metastatic stage, with the liver representing the main site of metastases (2). To date, the only curative treatment is the R0 resection of the primary tumor. However, even these patients often relapse and develop metastases shortly after or even during adjuvant therapy (3). Since PDAC patients with liver metastases have an even worse prognosis, it is of outmost importance to better understand the mechanisms underlying metastatic progression in the liver and to identify effective treatment options (4). Treatment with immune checkpoint inhibitors (ICI), e.g. targeting programmed death ligand 1 (PD-L1) or its receptor programmed cell death protein 1 (PD-1), have improved the therapy of many cancers but failed in PDAC yet. Monoclonal antibodies such as Durvalumab and Pembrolizumab blocking PD-L1 and PD-1, respectively, aim to boost cancer-directed immunity by induction of CD8^+^ T cell-mediated killing of tumor cells. However, so far ICI did not add any considerable benefit to the treatment of PDAC patients (5, 6). Nevertheless, a high infiltration of CD8^+^ T cells is associated with longer overall survival of PDAC patients indicating that CD8^+^ T cells exhibit a potent anti-tumorigenic potential in PDAC (5, 7). Moreover, absent PD-L1 expression and high CD8^+^ T cell infiltration of PDAC are even linked to a better prognosis (8). Of note, PD-L1 expression in PDAC is very heterogeneous, as some tumors show no PD-L1 expression while others show high expression (8, 9). In the latter cases, PD-L1 is mostly expressed by stromal cells rather than tumor cells (9). However, whether and how stromal cell-associated PD-L1 expression impacts immune evasion is still poorly understood.

Desmoplasia, the tumor-associated excessive formation of connective tissue formed by fibroblasts, stellate cells, and their activated counterparts, the myofibroblasts, represents a hallmark of PDAC (10). Desmoplasia is an integral component of the stroma of primary PDAC as well as its liver metastases. Hepatic stellate cells (HSC) make up to 5% of all liver cells and are important to maintain liver homeostasis. In the presence of Transforming Growth Factor-beta 1 (TGF-β1), Platelet-derived Growth Factor (PDGF), or Interleukin-6 (IL-6) HSC transdifferentiate into hepatic myofibroblasts (HMF). HMF are characterized by a high expression of alpha-smooth muscle actin (αSMA) as well as the secretion of various inflammatory mediators and ECM molecules, that have been reported to support tumor growth (11, 12). Furthermore, cancer-associated fibroblasts (CAF) can impact the immune response by secreting cytokines, like IL-6, Granulocyte Colony-stimulating Factor (G-CSF), and Macrophage Colony-stimulating Factor (M-CSF), or inhibiting CD8^+^ T cells by either expressing PD-L1 or promoting expression of PD-1 and cytotoxic T-lymphocyte-associated protein 4 (CTLA-4) on CD8^+^ T cells (13). Lenk et al. showed that the inflammatory status of the liver microenvironment is an essential driver for the outgrowth of liver metastases. Here, HMF promoted PDAC cell growth in a Vascular Endothelial Growth Factor (VEGF) dependent manner (14). Owing to their ability to release high amounts of ECM proteins and immune regulatory mediators, HMF generate a dense stroma, which acts as a physical barrier for immune cells and drugs (12). Although several studies have already demonstrated that myofibroblasts essentially contribute to drug resistance and immune evasion in PDAC (15–17), their impact on immune evasion of PDAC liver metastases is still poorly understood. Since metastases are not routinely resected in PDAC patients, tissue samples from PDAC metastases are rare and the expression of immune regulatory molecules such as PD-L1 within liver metastases is rarely characterized. Hence, this study intended to elucidate the role of PD-L1 in the interplay of PDAC cells, the hepatic microenvironment, and CD8^+^ T lymphocytes in order to provide novel insight into the mechanisms contributing to immune escape of PDAC liver metastases.

## 2 Material and methods

### 2.1 Immunohistochemical analysis of liver tissues with PDAC metastases

Formalin-fixed and paraffin embedded (FFPE) biopsy samples or diagnostic resection specimens from liver metastases of 15 PDAC patients were used for immunohistochemical (IHC) analyses. The study was approved by the ethics committee of the Medical Faculty of Kiel University (reference number: A110/99). Written informed consent was obtained from all patients. Thirteen patients were male and two were female. The median age at diagnosis was 70 (range: 59-82). The histopathological diagnosis was confirmed by board certified surgical pathologists. From one patient a histopathological assessment was also performed from the primary tumor, from all other patients only from the liver biopsies. One patient suffered from metastasized PDAC with liver metastases and peritoneal carcinosis. Liver tissues for sectioning were mostly obtained from liver biopsies. Serial sections were used throughout this study and stained with antibodies directed against Pan-Cytokeratin (PanCK) (dilution 1:200, clone AE1/AE3, NeoMarkers *via* ThermoFisher Scientific, Waltham, MA, USA), αSMA (dilution 1:400, clone 1A4, NeoMarkers *via* ThermoFisher Scientific, Waltham, MA, USA), CD8 (dilution 1:100, clone C8144B, Leica Biosystems GmbH, Wetzlar, Germany), and PD-L1 (dilution 1:100, clone: E1L3N, Cell Signaling Technology, Danvers, MA, USA).

Furthermore, IHC double staining of liver metastases was performed. The first step involved the staining of PD-L1 (dilution 1:100, clone E1L3N; Cell Signaling Technology, Danvers, MA, USA). Antigen retrieval was achieved with ER2 (EDTA-buffer Bond pH 9.0; 20 minutes). The antigen retrieval step was modified for the PD-L1 staining of those slides, which were to be combined with αSMA staining in the second step. In those cases, PD-L1 visualization was enhanced in relation to the naturally intense αSMA signal by prolonging ER2 antigen retrieval to 30 minutes. The immunoreaction was visualized with the Bond™ Polymer Refine Detection Kit (DS 9800; brown labeling; Novocastra; Leica Biosystems GmbH, Wetzlar, Germany) resulting in a brown color. The second step involved the staining of either αSMA (dilution 1:400, clone: 1A4, NeoMarkers *via* Thermo Fisher Scientific, Waltham, MA, USA), or CD68 (dilution 1:100, clone: 514H12, Leica Biosystems GmbH, Wetzlar, Germany), or PanCK (dilution 1:200, clone AE1/AE3, NeoMarkers *via* ThermoFisher Scientific, Waltham, MA, USA). Antigen retrieval was carried out with ER1 (citrate buffer Bond pH 6.0; 20 minutes for αSMA), or ER2 (EDTA-buffer Bond pH 9.0; 20 minutes for CD68). The immunoreaction was visualized with the BOND™ Polymer Refine Red Detection Kit (DS9390; red labeling; Leica Biosystems GmbH, Wetzlar, Germany) resulting in a red color.

The IHC stainings were carried out on the autostainer BOND™ RX system (Leica Biosystems GmbH, Wetzlar, Germany). The stained tissue sections were scanned on the Hamamatsu NanoZoomer 2.0 RS digital slide scanner (Hamamatsu Photonics, Shizuoka Prefecture, Japan). Scanned tissue sections were analyzed with NDP.view2 software (Hamamatsu Photonics, Shizuoka Prefecture, Japan).

First, PanCK staining of the liver sections were used to categorize metastases into small (≤ 1500 µm) and large (> 1500 µm) metastases. Afterwards, the predominant localization of CD8, αSMA, and PD-L1 staining was rated as followed i) mostly in the tumor center, ii) mostly at the invasion front, or iii) evenly distributed between both regions. The analysis was performed at a magnification of 5-fold or 10 fold depending on the size of the respective metastasis. Further, the proportion of cells stained for CD8 and PD-L1 was analyzed in 10 representative fields of view (FoV) both at the invasion front of the metastases and in the tumor center at 100-fold magnification. The scoring was graded as i) 0% (negative), ii) ≤ 1%, or iii) > 1%. Additionally, PD-L1 staining intensity was scored as absent, low, or high. Finally, the percentages of the proportion of each score were calculated. All stainings were evaluated independently by two examiners (SB and LA).

### 2.2 Cell lines and cell culture

Human PDAC cell lines PancTu1 and Panc89 were cultured in PDAC cell medium (RPMI 1640 medium supplemented with 10% FCS, 1% L-Glutamine, and 1% sodium pyruvate (PAN-Biotech GmbH, Aidenbach, Germany)). PancTu1 cells were isolated from a primary tumor of a female PDAC patient and were used as a PDAC cell line with moderate PD-L1 cell surface expression. PancTu1 cells exhibit mutations in *k-ras* (G12V) and *p53* (C176S) and a depletion of *p16*, while *SMAD4* shows a wildtype status (18, 19). Panc89 cells were isolated of a lymph node metastasis of a 64-year-old male PDAC patient and were used as a PDAC cell line with low PD-L1 surface expression. Panc89 cells exhibit a mutation in *p53* (T220C) and depletion of *p16*, while the genes *k-ras* and *SMAD4* show a wildtype status (18, 19). Human myofibroblasts (Provitro GmbH, Berlin, Germany) were cultured in stellate cell medium supplemented with 2% FCS, 1% stellate cell growth supplement, and 1% Penicillin and Streptomycin (Science Cell Research Laboratories, Carlsbad, CA, USA). One ng/ml of recombinant human TGF-β1 (BioLegend, San Diego, CA, USA) was added to maintain the myofibroblastic phenotype. All cell lines were cultivated in a 75 cm^2^ cell culture flask at 37°C, 5% CO_2_, and 86% relative humidity. The cells were regularly examined with a MycoAlert™ PLUS Mycoplasma Detection Kit (Lonza Group, Basel, Switzerland) to assure mycoplasma-free conditions.

### 2.3 Isolation of primary human CD8^+^ T cells

Peripheral blood mononuclear cells (PBMC) were isolated from blood donations by healthy donors provided by the Institute of Transfusion Medicine, UKSH Campus Kiel. The research was approved by the ethics committee of the Medical Faculty of Kiel University and the University Hospital Schleswig-Holstein, Campus Kiel (reference number: A110/99 and D490/17). Written informed consent from all donors was obtained. For PBMC isolation, a Pancoll (PAN-Biotech GmbH, Aidenbach, Germany) density gradient centrifugation (350 xg, 25 min, 4°C) was performed. Afterwards, 125x10^6^ isolated PBMCs suspended in 10 ml RPMI 1640 medium supplemented with 1% FCS were transferred into a 75 cm^2^ cell culture flask. After 45 minutes, the supernatant was carefully removed to obtain only the lymphocytes. Only lymphocyte purities of over 80% were used for isolation of CD8^+^ T cells, which were verified by flow cytometry. In order to obtain untouched CD8^+^ T cells, magnetic cell sorting was performed using a negative selection strategy with the CD8^+^ T cell isolation kit from Miltenyi Biotec, according to the manufacturer’s instructions (Miltenyi Biotec GmbH, Bergisch Gladbach, Germany).

### 2.4 Activation of CD8^+^ T cells

Activation of primary naïve CD8^+^ T cells was performed by stimulation with anti-CD3 and anti-CD28 antibodies which were used to mimic antigen-presenting cells. For this purpose, a 24-well plate was coated with 1.5 µg/ml anti-CD3 antibody (clone: OKT3, BioLegend, San Diego, CA, USA) diluted in 200 µl sterile PBS for 2 h at 37°C. Afterwards, the plate was washed twice with PBS to remove all unbound antibodies. Then, 1.5 x10^6^ CD8^+^ T cells per well were seeded in 1 ml PDAC cell medium further supplemented with 2% HEPES and 1% Penicillin and Streptomycin (TCM). Finally, 1.5 µg/ml anti-CD28 antibody (clone: CD28.2, BioLegend, San Diego, CA, USA), and 60 ng/ml recombinant human IL-2 (BioLegend, San Diego, CA, USA) were added. After 72 h, CD8^+^ T cells were collected and used for subsequent coculture experiments.

### 2.5 Spheroid cultures of PDAC cells and HMF

In order to mimic the tumor cell to HMF ratio in small and large PDAC liver metastases, different ratios of PancTu1 or Panc89 cells and HMF were seeded for spheroid formation. Here, a PDAC cell to HMF ratio of 3:1 was seeded to mimic large metastases. A PDAC cell to HMF ratio of 5:1 was seeded to mimic small metastases. HMF and either PancTu1 or Panc89 cells were seeded together at the respective ratios at a total cell number of 2x10^4^ in 150 µl TCM in 96-well ultra-low attachment plates (faCellitate, Mannheim, Germany). As control, PancTu1 and Panc89 cells, respectively, were seeded as monoculture spheroids. After seeding, plates were centrifuged at 300 xg for 5 min and spheroids were cultured for 72 h, at 37°C, 5% CO2, and 86% relative humidity.

### 2.6 Spheroid cultures with CD8^+^ T cells

In order to investigate the effect of PDAC cells and different amounts of HMF on the effector phenotype of CD8^+^ T cells, medium from spheroid cultures was removed after 48 h and 2x10^5^ activated CD8^+^ T cells were added in 150 µl of TCM/well for further 24 h.

### 2.7 Treatments of spheroid cultures

For ICI treatment medium of spheroid cultures was discarded after 48 h. Then, 2x10^5^ activated CD8^+^ T cells were added and spheroid cocultures were treated with either 10 µg/ml of the respective isotype control antibody [hIgG1 (AstraZeneca, Cambridge, UK)/hIgG4 (Merck, KGaA, Darmstadt, Germany)], 10 µg/ml Durvalumab (AstraZeneca, Cambridge, UK), or 10 µg/ml Pembrolizumab (MSD, Kenilworth, NJ, USA) for 24 h.

Treatment with 10 µg/ml Gemcitabine (Hexal, Holzkirchen, Germany) was conducted 24 h after seeding of the spheroids. Respective controls were left untreated. After 24 h of treatment, the medium was discarded and 2x10^5^ activated CD8^+^ T cells were added for further 24 h.

### 2.8 Flow cytometry

The expression of cell surface proteins on PDAC, HMF, and CD8^+^ T cells was examined by immunofluorescence staining and subsequent flow cytometric analysis. For staining, supernatants containing CD8^+^ T cells were collected from spheroid cultures and spheroids were mechanically dissociated with a 30G cannula to generate a single-cell suspension. Staining was performed according to the protocol of BioLegend. PancTu1, Panc89 cells, and HMF were stained with anti-PD-L1-PeCy7 antibody (clone: MIH3, #374506). PDAC cells were also stained with anti-EpCAM-APC antibody (clone: 9c4, #324208 (all from BioLegend, San Diego, CA, USA). CD8^+^ T cells were stained with anti-CD8-FITC (clone: RPA-T8, #301006), anti-PD-1-PE (clone: EH12.2H7, #621608), anti-CD69-PeCy7 (clone: FN50, #310912), and anti-CD25-APC antibodies (clone: BC96, #302610) (all from BioLegend, San Diego, CA, USA). Staining specificity was verified by staining with respective isotype control antibodies: mIgG1-PeCy7 (clone: MOPC-21, #400126), mIgG2b-APC (clone: MPC-11, #400321), mIgG1-FITC (clone: MOPC-21, #400108), mIgG1-PE (clone: MOPC-21, #400112), and mIgG1-PAC (clone: MOPC-21, #400122), (all from BioLegend, San Diego, CA, USA)). For relative quantification of cell surface expression levels, median fluorescence intensity (MFI) ratio was calculated by dividing the MFI detected for the specific staining by the MFI detected for the staining with the respective isotype controls. For the assessment of the lymphocyte purity prior to isolation of CD8^+^ T cells (see section Isolation of primary human CD8^+^ T cells) cells were washed once with MACS Buffer (PBS (PAN-Biotech GmbH, Aidenbach, Germany) supplemented with 0.5% BSA (Biomol, Hamburg, Germany) and 2 mM EDTA (Carl Roth GmbH, Karlsruhe, Germany)) and then fixed in MACS Buffer supplemented with 1% PFA (ThermoFisher Scientific, Waltham, MA, USA). Data acquisition was performed with a MACS Quant X (Miltenyi Biotec GmbH, Bergisch Gladbach, Germany) and evaluation was conducted using the FlowJo program v10.8.1 (BD Bioscience, Franklin Lakes, New Jersey, USA).

### 2.9 LEGENDplex™

LEGENDplex™ Human CD8/NK Mix and Match Subpanel and LEGENDplex™ Human total TGF-β1 (BioLegend, San Diego, CA, USA) were used according to the manufacturer’s instructions for quantification of Interferon-gamma (IFN-γ), Granzyme A, Granzyme B, Perforin, Granulysin, and TGFβ-1 concentrations in cell culture supernatants. Measurement was performed on the BD FACSymphony™ A1 flow cytometer (BD Bioscience, Franklin Lakes, New Jersey, USA). The evaluation was conducted using the LEGENDplex™ data analysis software (BioLegend, San Diego, CA, USA).

### 2.10 M30 CytoDeath™ ELISA

Cell death induction in PDAC cells in the different spheroid cultures was assessed with the M30 CytoDeath ELISA kit following the manufacturer’s instructions (Diapharma Group Inc., West Chester, OH, USA). The assay detects caspase-cleaved Keratin 18 (ccK18) which is generated exclusively by PDAC cells undergoing apoptotic cell death in the used spheroid cultures. Measurement of ccK18 in supernatants of spheroid cultures was performed on the Infinite 200 PRO microplate reader (Tecan, Crailsheim, Germany).

### 2.11 Statistical analysis

Statistical analyses were performed with GraphPad Prism 9.2.0 (GraphPad Software, San Diego, CA, USA). First, data was tested for normal distribution and equal variances using Shapiro-Wilk test. Two tailed t-test was performed for analysis of two normally distributed data sets. Groups of data sets that did not pass the test for normal distribution and equal variances were analyzed by the Mann-Whitney Rank Test. Parametric data sets of including more than two groups were analyzed with the one-way-analysis of variance (one-way-ANOVA). For multiple comparison the Tukey Test was used. Non-parametric data sets comprising more than two groups were analyzed using Kruskal-Wallis one-way-ANOVA on Ranks and for multiple comparison the Dunn’s Test was performed. Statistical significance was considered for p-values of < 0.05, according to Student-Newman-Keuls test for parametric and Dunn method for non-parametric data. The significance levels are indicated by asterisks: p < 0.05 = *, p < 0.01 = **, p < 0.001 = ***.

## 3 Results

### 3.1 Different spatial distribution of myofibroblasts, CD8^+^ T cells, and PD-L1 expression in small and large liver metastases of PDAC patients

**Figure 1:**
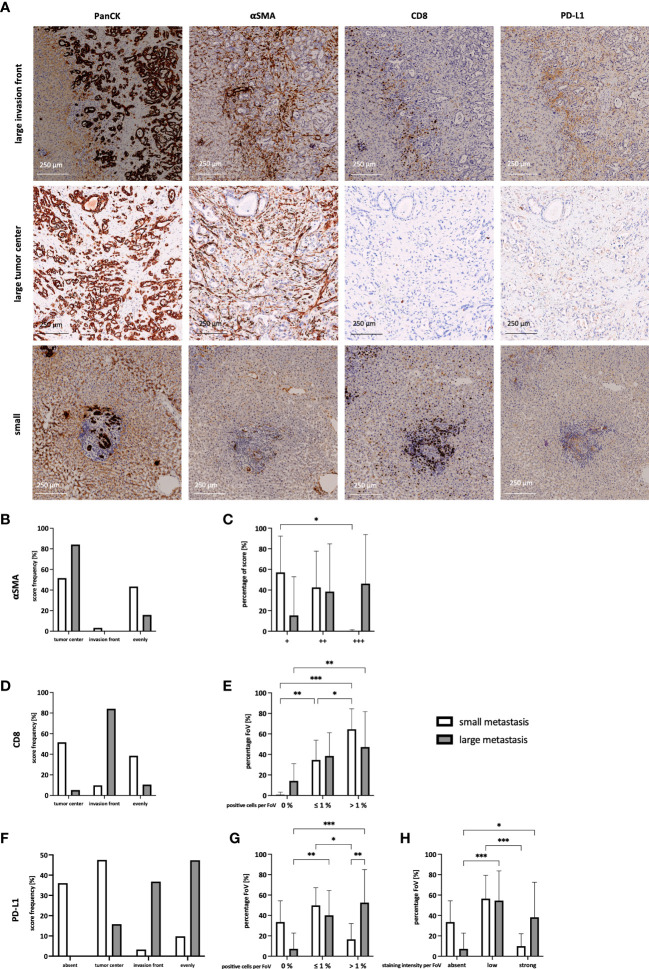
Different spatial distribution of myofibroblasts, CD8^+^ T cells, and PD-L1 expression in small and large liver metastases of PDAC patients. **(A)** Representative images of the staining of PanCK, αSMA, CD8 and PD-L1 in large and small liver metastases of PDAC patients. The main localization of **(B)** αSMA, **(D)** CD8 T cells, and **(F)** PD-L1 expression were scored comparing absent expression and expression in the tumor center, at the invasion front, or evenly distributed between tumor center and invasion front in small and large liver metastases. **(C)** The abundance of αSMA was rated as low number (+), medium number (++), or higher number (+++) of myofibroblasts in each metastasis. Furthermore, a maximum of 10 microscopic Fields of View (FoV) in the tumor center and at the invasion front were analyzed and the proportion of **(E)** CD8^+^ T cells and **(G)** PD-L1^+^ cells was determined. The proportion was ranked as 0%, ≤ 1%, or > 1% for CD8 and PD-L1, respectively. **(H)** Intensity of PD-L1 staining was ranked for each FoV, comparing absent, low, or strong expression. Data represents the mean ± SD of 15 independent liver tissue sections. * = p < 0.05, ** = p < 0.01, ***p < 0.001.

To better understand whether PD-L1 might play a role in immune evasion of PDAC cells in liver metastasis, it was examined whether PD-L1 is expressed in liver metastasis, and how this relates to defined stroma compositions. For this purpose, serial sections of liver metastases from 15 PDAC patients were stained for PanCK, αSMA, CD8, and PD-L1 (**Figure 1A**). Since previous studies reported that stroma composition markedly differs with regard to metastasis size (20), metastases were categorized into small (≤ 1500 µm) and large (> 1500 µm) metastases (**Supplementary Figure 1A**). In general, more small than large metastases were detected in the liver sections (**Supplementary Figure 1B**). First, desmoplasia was assessed by evaluating the abundance of myofibroblasts. Small and large metastases clearly differ with respect to aSMA+ the spatial distribution (**Figure 1B**) and extent (**Figure 1C**) of αSMA+ myofibroblasts. While in small metastases αSMA-expression was detectable in the tumor center or evenly distributed between tumor center and the invasion front, αSMA staining was predominantly located inside of large metastases (**Figure 1B**). Further, the number of myofibroblasts was rated and compared (+ = low number, ++ = medium number, or +++ = high number of myofibroblasts). While small metastases were mostly characterized by low numbers of myofibroblasts, 50% of large metastases exhibited high numbers (**Figure 1C**). Moreover, also spatial distribution of CD8^+^ T cells clearly differed between small and large metastases. While CD8^+^ T cells were predominantly located inside or evenly distributed in small metastases, in large metastases the majority of CD8^+^ T cells were almost exclusively found at the invasion front (**Figure 1D**). In line with these findings, 15% of all FoV examined in the tumor center of large metastases exhibited no CD8^+^ T cells. These FoV were only scored in the tumor centers (**Supplementary Figure 1C**). However, within the majority of FoV more than 1% of these cells were stained for CD8 in both small and large metastases, albeit in small metastases over 60% of FoV contained even higher proportions of CD8^+^ T cells (**Figure 1E**). Finally, histoanatomical localization and intensity of PD-L1 staining were analyzed. Here, 35% of small metastases showed no PD-L1 staining and if present, staining was predominantly observed in the tumor center. In contrast, PD-L1 staining in large metastases was either evenly distributed or localized predominantly at the invasion front (**Figure 1F**). Of note, FoV in large metastases exhibited significantly more PD-L1^+^ cells compared to small metastases (**Figure 1G**) which were associated with significantly more FoV with strong PD-L1 staining. Interestingly, strong PD-L1 staining was mostly found at the invasion front (**Figure 1H**, **Supplementary Figure 1E**). In contrast, PD-L1^+^ cells were detected only in 33% of all FoV in small metastases. Comparing the spatial distribution of PD-L1, PanCK, and αSMA in serial liver sections demonstrated that PD-L1 staining was mainly colocalized with αSMA staining rather than PanCK staining, indicating that PD-L1 is mainly expressed by myofibroblasts in PDAC liver metastases (**Figure 1A**). For validation, IHC double stainings of PanCK/PD-L1, αSMA/PD-L1, and CD68/PD-L1 were performed showing that PD-L1 was more expressed by myofibroblasts and also macrophages rather than tumor cells (**Supplementary Figures 2A-C**).

In summary, our IHC analyses revealed that small liver metastases comprise more CD8^+^ T cells in their tumor center with no or low PD-L1 expression, while large metastases exhibit stronger PD-L1 expression and less CD8^+^ T cells being mostly located at the invasion front. Furthermore, HMF represent an abundant stroma population in liver metastases of PDAC, with clear differences in the spatial distribution in small and large metastases and being a main source of PD-L1 expression. Overall, these findings support the hypothesis that outgrowth of hepatic PDAC metastases is accompanied by stroma remodeling involving considerable alterations in the localization and number of CD8^+^ T cells, myofibroblasts as well as PD-L1 expression.

### 3.2 Spheroid coculture of PDAC cells and HMF mimic hepatic PDAC metastases

**Figure 2:**
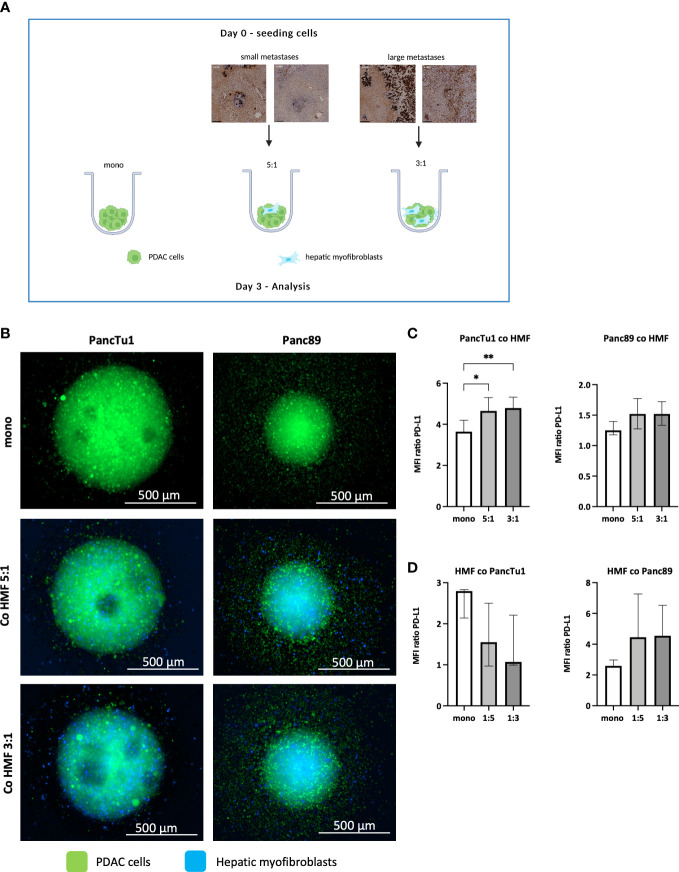
Spheroid coculture of PDAC cells and HMF mimic hepatic PDAC metastases. **(A)** Schematic illustration of the PDAC spheroids cultures: PancTu1 and Panc89 cells were either seeded in mono- or coculture with hepatic myofibroblasts (HMF) at different ratios (5:1 or 3:1) in ultra-low attachment plates for 3 days. Figure was created with BioRender.com. **(B)** Representative images of PancTu1 and Panc89 spheroids in mono- and coculture with HMF after 3 days. In green (cell tracker green) PDAC cells and in blue (cell trace violet) HMF. For flow cytometry analysis, spheroids were mechanically dissociated and the median fluorescence intensity (MFI) ratio of PD-L1 on **(C)** PancTu1 cells, Panc89 cells, and **(D)** HMF after mono- or coculture was determined. Data represents the mean ± SD (normally distributed) or the median with interquartile range (not normally distributed). N=5. * = p < 0.05, ** = p < 0.01.

The IHC analysis of PDAC liver metastases revealed clear differences with respect to PD-L1 expression and number of HMF in small and large metastases. To investigate whether the PD-1/PD-L1 axis and HMF contribute to immune escape of PDAC liver metastases, a stroma enriched 3D spheroid coculture model was established *in vitro*. To mimic small and large hepatic PDAC metastases, PancTu1 or Panc89 PDAC cells and HMF were seeded at different ratios (PDAC : HMF 5:1 = 83,3% vs. 16.7%, representative for small metastases; PDAC : HMF 3:1 = 75% vs. 25%, representative for large metastases). As control, PancTu1 and Panc89 cell monoculture spheroids were seeded (**Figure 2A**). In general, mono- and coculture spheroids of PancTu1 cells were larger (~800 µm) compared to Panc89 cell spheroids (~500 µm). In line with the *in situ* findings of small and large liver metastases, less HMF were present at the 5:1 ratio (small metastases) compared to the 3:1 ratio (large metastases) in both PDAC cell models after spheroid formation. In addition, HMF appeared to be evenly distributed in the spheroids at a 5:1 ratio and more inside at a 3:1 ratio (**Figure 2B**). Moreover, monocultured PancTu1 cells exhibited higher PD-L1 cell surface levels compared to Panc89 cells, which well mimics the expression heterogeneity in tumor cells observed in hepatic PDAC metastases. After coculture, PD-L1 cell surface levels were slightly increased in both PDAC cell lines in the presence of HMF independent of the coculture condition (**Figure 2C**). In line with the *in situ* findings, monocultured HMF exhibited higher PD-L1 cell surface levels compared to those on PDAC cells which were slightly downregulated by coculture with PancTu1 cells and slightly upregulated by coculture with Panc89 cells (**Figure 2D**).

Overall, our stroma enriched spheroid coculture model containing PDAC cells and HMF at different ratios well mimicked the proportion and distribution of PDAC cells and HMF as well as their PD-L1 expression detected in small and large hepatic PDAC metastases.

### 3.3 The effector phenotype of CD8^+^ T cells and induction of PDAC cell death are impaired by HMF in PDAC coculture spheroids

**Figure 3:**
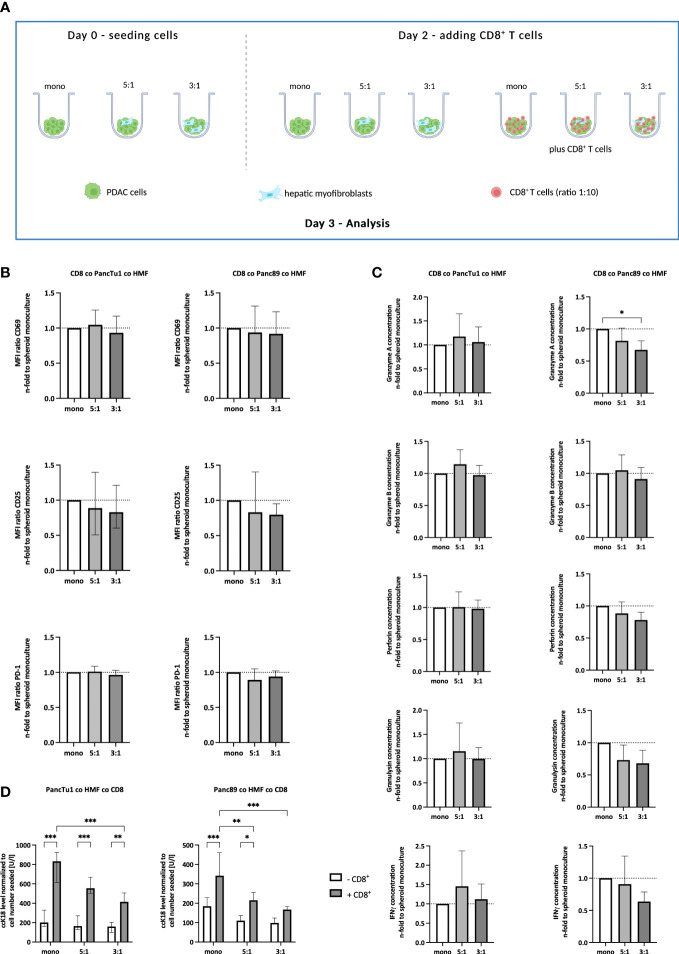
The effector phenotype of CD8^+^ T cells and induction of PDAC cell death are impaired by HMF in PDAC coculture spheroids. **(A)** Schematic illustration of the PDAC spheroids cultures in the absence or presence of CD8^+^ T cells. PancTu1 and Panc89 cells were either seeded in mono- or coculture with hepatic myofibroblasts (HMF) at different ratios (5:1 or 3:1) in ultra-low attachment plates. After 48 h, activated CD8^+^ T cells were added at a ratio of 1:10 in one half of the cultures for further 24 h. Figure was created with BioRender.com. **(B)** CD8^+^ T cells obtained from different spheroid cultures were stained for CD69, CD25, and PD-1 analyzed by flow cytometry analysis and the median fluorescence intensity (MFI) ratio of each specific staining was determined. **(C)** The concentration of Granzyme A, Granzyme B, Perforin, Granulysin, and IFNγ was analyzed in the supernatants of differentially cultured spheroids. Data was normalized on the expression and the concentration in supernatants of CD8^+^ T cells in culture with mono-culture spheroids. **(D)** Levels of caspase-cleaved cytokeratin 18 (ccK18) were measured in supernatants of mono- and coculture spheroids in the absence or presence of CD8^+^ T cells and normalized to the seeded PDAC cell number. Data represents the mean ± SD (normally distributed) or the median with interquartile range (not normally distributed). N=4. * = p < 0.05, ** = p < 0.01, ***p < 0.001.

Having provided evidence that our *in vitro* model well simulates the contextual situation identified in PDAC liver metastases *in situ*, we next examined whether the effector phenotype of CD8^+^ T cells is impacted by the different stroma conditions. For this purpose, CD8^+^ T cells were pre-activated for 72 hours and then seeded in the different spheroid cultures (**Figure 3A**). At this time point, CD8^+^ T cells exhibited high cell surface expression of CD25 and the early activation marker CD69 along with considerable expression levels of PD-1 (data not shown). As seen in **Figure 3B**, cell surface levels of CD25 and CD69 as well as PD-1 in CD8^+^ T cells were hardly affected by different spheroid coculture conditions in both PDAC cell models. Next, supernatants derived from CD8^+^ T cells cultured with mono- and coculture PDAC cell spheroids were analyzed for the presence of cytotoxic effector molecules (**Figure 3C**). Levels of Granzyme A and B, Perforin, Granulysin, and IFNγ were not altered in supernatants obtained from CD8^+^ T cells cultured with HMF-enriched PancTu1 spheroids compared to supernatants from PancTu1 monoculture spheroids. However, levels of Granzyme A, Granulysin, and IFNγ were decreased in supernatants from CD8^+^ T cells after culture with Panc89 HMF spheroids compared to culture with Panc89 monoculture spheroids being even more reduced in the presence of higher amounts of HMF (PDAC : HMF ratio 3:1) resembling large metastases (**Figure 3C**). Finally, we analyzed whether the effector phenotype of CD8^+^ T cells correlates with the induction of PDAC cell death. For this purpose, supernatants of CD8^+^ T cells cultured with PDAC mono- and coculture spheroids were analyzed for the presence of ccK18 indicative for induction of PDAC cell death. As shown in **Figure 3D**, ccK18 levels were detectable at low levels and comparable between mono- and coculture PancTu1 and Panc89 spheroids in the absence of CD8^+^ T cells. In the presence of CD8^+^ T cells, significantly elevated ccK18 levels were measured in both PancTu1 and Panc89 spheroids devoid of HMF. However, a clear reduction of ccK18 levels was observed in supernatants of coculture spheroids of either PDAC cell line in the presence of CD8^+^ T cells, with the strongest reduction observed in supernatants of spheroids containing higher amounts of HMF (3:1). This effect was more pronounced in the Panc89 HMF spheroids being in line with more pronounced reduction of CD8^+^ T cell effector molecules in supernatants from HMF enriched Panc89 coculture spheroids (**Figure 3D**).

Overall, these data suggest that HMF impair the release of effector molecules by CD8^+^ T cells and the induction of PDAC cell death, an effect that is dependent on the amount of HMF but not on the PDAC cells.

### 3.4 ICI treatment leads to elevated secretion of distinct CD8^+^ T cell effector molecules but does not increase PDAC cell death

**Figure 4:**
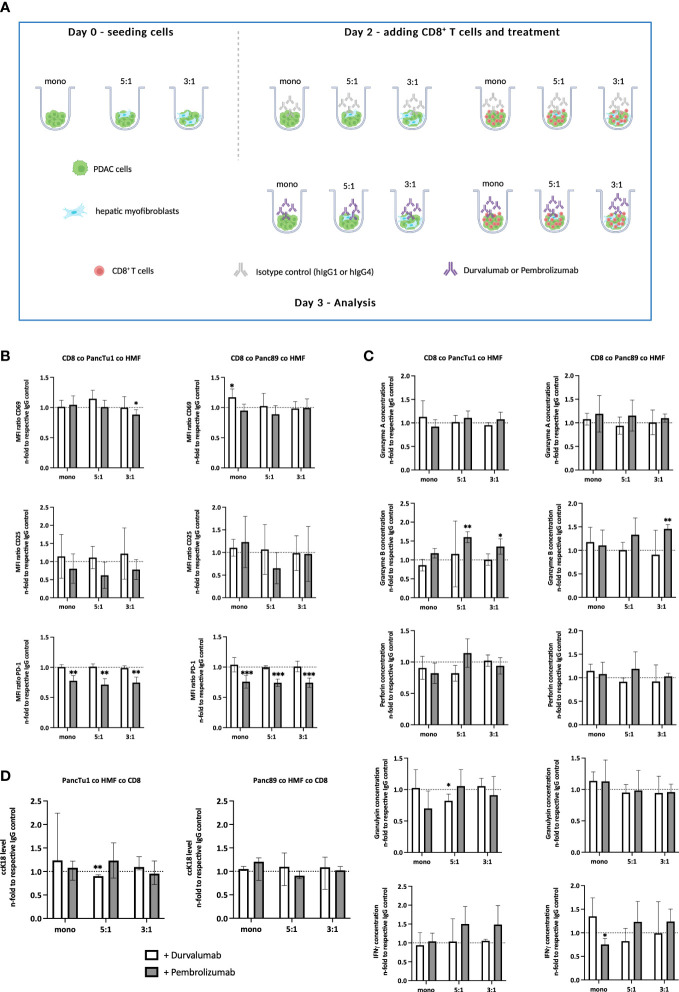
ICI treatment leads to elevated secretion of distinct CD8^+^ T cell effector molecules but does not increase PDAC cell death. **(A)** Schematic illustration of the PDAC spheroids cultures before adding CD8^+^ T cells. PancTu1 and Panc89 cells were either seeded in mono- or coculture with hepatic myofibroblasts (HMF) at different ratios (5:1 or 3:1) in ultra-low attachment plates. After 48 h, the medium was discarded and activated CD8^+^ T cells were added at a ratio of 1:10 in one half of the cultures and either 10 µg/ml of the respective isotype control, Durvalumab or Pembrolizumab were applied for 24 h. Figure was created with BioRender.com. **(B)** CD8^+^ T cells obtained from different spheroid cultures were stained for CD69, CD25, and PD-1 by flow cytometry analysis where the median fluorescence intensity (MFI) ratio of each specific staining was determined. **(C)** The concentration of Granzyme A, Granzyme B, Perforin, Granulysin, and IFNγ was analyzed in the supernatants of differentially treated culture spheroids. Data was normalized on the expression and on the concentration in supernatants of the respective IgG control treated spheroid cultures. **(D)** Levels of caspase-cleaved cytokeratin 18 (ccK18) were measured in supernatants of CD8^+^ T cells with mono- and coculture spheroids after ICI treatment. Data was normalized to the isotype control of the respective spheroid culture setting. Data represents the mean ± SD (normally distributed) or the median with interquartile range (not normally distributed). N=4. * = p < 0.05, ** = p < 0.01, ***p < 0.001.

After having shown that the PDAC-HMF interplay impairs the effector phenotype of CD8^+^ T cells, it was investigated whether ICI treatment is able to restore the effector phenotype and leads to enhanced induction of PDAC cell death. PD-L1 and PD-1 were blocked by Durvalumab and Pembrolizumab, respectively, for 24 hours under the distinct spheroid conditions cultured with CD8^+^ T cells (**Figure 4A**). Successful blocking of cell surface associated PD-L1 and PD-1 by either Durvalumab or Pembrolizumab treatment on each cell population was validated by flow cytometry. Cell surface levels of PD-1 on CD8^+^ T cells were significantly lower after Pembrolizumab treatment (**Figure 4B**) and cell surface levels of PD-L1 on PDAC cells and HMF were also significantly lower after Durvalumab treatment compared to treatment with respective the control antibody (**Supplementary Figures 3A-C**). In order to examine the effect of Durvalumab and Pembrolizumab treatment on the respective effector phenotype of CD8^+^ T cells, we first analyzed cell surface expression levels of T cell activation markers CD25 and CD69. No considerable effects on the cell surface expression levels of CD69 and CD25 on CD8^+^ T cells by Durvalumab treatment after culture with either spheroid condition was observed (**Figure 4B**). However, treatment with Pembrolizumab led to a slight decrease of the expression of CD25 in CD8^+^ T cells cultured with PancTu1 coculture spheroids but not with Panc89 coculture spheroids, while the expression of CD69 was only decreased on CD8^+^ T cells when cultured with PancTu1 coculture spheroids at a ratio of 3:1 (**Figure 4B**). Next, the concentration of CD8^+^ T cell effector molecules in supernatants of the different spheroid cultures was analyzed. Durvalumab did almost not affect levels of Granzyme A, Granzyme B, Perforin, Granulysin, and IFNγ in supernatants of CD8^+^ T cells cultured with either PDAC cell spheroids (**Figure 4C**, light grey), except Granulysin levels which were diminished after coculture with PancTu1:HMF spheroids (5:1 ratio). In contrast, levels of Granzyme B and IFNγ were elevated in supernatants from CD8^+^ T cells cultured with Panc89 coculture spheroids but not with Panc89 monoculture spheroids after Durvalumab treatment. Notably, no decrease in Granzyme A, Granulysin, and IFNγ concentration was detectable anymore in supernatants from Panc89 cell spheroids enriched with HMF (mainly at 3:1 ratio, **Figure 4B**, light grey), indicating a compensation of the HMF mediated inhibitory effect by the PD-L1 inhibitor. Pembrolizumab treatment did almost not affect levels of Granzyme A, Perforin, and Granulysin in supernatants of CD8^+^ T cells cultured with mono- or coculture spheroids of either PDAC cell line (**Figure 4C**, dark grey), while levels of Granzyme B and IFNγ were enhanced in supernatants of HMF enriched spheroids of PancTu1 and Panc89 cells. Finally, it was analyzed whether ICI treatment increased PDAC cell death under the different culture conditions. Overall, no considerable effect of PD-L1 or PD-1 blockade on PDAC cell death was detectable in either spheroid condition and Durvalumab treatment led even to decreased ccK18 levels in supernatants of CD8^+^ T cells and HMF-enriched PancTu1 spheroids (at 5:1 ratio, **Figure 4D**, light grey).

Overall, these data indicate that although either ICI treatment led to slight elevation of distinct CD8^+^ T cell cytotoxic effector molecules which seemed to be dependent on the presence of HMF and PDAC cells, this did not result in enhanced induction of PDAC cell death.

### 3.5 Gemcitabine affects the effector phenotype of CD8^+^ T cells and PDAC cell death induction in dependence on PDAC cells

**Figure 5:**
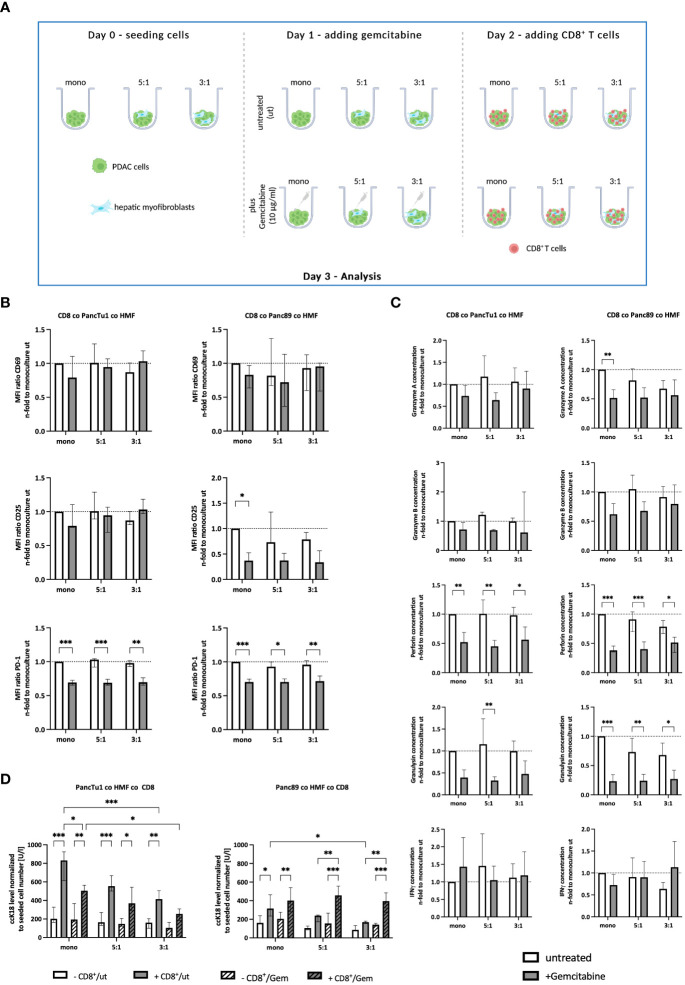
Gemcitabine affects the effector phenotype of CD8^+^ T cells and PDAC cell death induction in dependence on PDAC cells. **(A)** Schematic illustration of Gemcitabine treatment of PDAC spheroids cultures before adding CD8^+^ T cells. PancTu1 and Panc89 cells were either seeded in mono- or coculture with hepatic myofibroblasts (HMF) at different ratios (5:1 or 3:1) in ultra-low attachment plates for 24 h. Then, spheroids were either left untreated or treated with 10 µg/ml Gemcitabine. After 24 h, the medium was changed and activated CD8^+^ T cells were added at a ratio of 1:10 in one half of the cultures. **(B)** CD8^+^ T cells obtained from different spheroid cultures were stained for CD69, CD25, and PD-1 by flow cytometry analysis and the median fluorescence intensity (MFI) ratio of each specific staining was determined. **(C)** The concentration of Granzyme A, Granzyme B, Perforin, Granulysin, and IFNγ was analyzed in supernatants of differentially and treated culture spheroids. Data was normalized on the expression and on the concentration in supernatants of CD8^+^ T cells in culture with untreated mono-cultured spheroids. **(D)** Levels of caspase-cleaved cytokeratin 18 (ccK18) were measured in supernatants of CD8^+^ T cells with untreated or Gemcitabine treated mono- and coculture spheroids. The ccK18 levels were normalized to seeded cell number of PDAC cells. Data represents the mean ± SD (normally distributed) or the median with interquartile range (not normally distributed). N=4. * = p < 0.05, ** = p < 0.01, ***p < 0.001.

Since treatment with Gemcitabine represents the most frequent first-line therapy of PDAC patients, it was investigated whether Gemcitabine impacts PD-L1 and PD-1 expression as well as the effector phenotype of CD8^+^ T cells in the context of the PDAC-HMF interplay. For this purpose, monocultured and HMF-enriched PancTu1 and Panc89 spheroids were either left untreated or treated with Gemcitabine for 24 hours and then cultured with CD8^+^ T cells (**Figure 5A**). As shown in **Figure 5B**, Gemcitabine treatment led to diminished CD25 cell surface expression levels on CD8^+^ T cells derived from Panc89 mono- and coculture spheroids, while CD69 cell surface expression was almost not altered on CD8^+^ T cells obtained from either PDAC cell spheroid culture (**Figure 5B**). PD-1 cell surface levels were significantly lower on CD8^+^ T cells derived from any culture with PDAC mono- or coculture spheroids, which were pretreated with Gemcitabine (**Figure 5B**). In contrast, Gemcitabine hardly altered PD-L1 cell surface expression on monocultured PDAC cells as well as HMF (**Supplementary Figure 4**). Moreover, levels of Granzyme A, Granzyme B, Perforin, and Granulysin were reduced – either by trend or significantly - in all supernatants of CD8^+^ T cells and Gemcitabine treated PDAC mono- and coculture spheroids, while IFNγ was almost not altered in supernatants of Gemcitabine treated cultures (**Figure 5C**). Finally, it was analyzed whether Gemcitabine affects induction of PDAC cell death in dependence on the presence of HMF and CD8^+^ T cells. While treatment with Gemcitabine hardly impacted cell death of either PDAC cell line in mono- and coculture spheroids in the absence of CD8^+^ T cells, clear differences were observed in their presence (**Figure 5D**). While ccK18 levels were markedly lower in supernatants of CD8^+^ T cells cultured with Gemcitabine treated PancTu1 mono- and coculture spheroids, elevated ccK18 levels were noted in supernatants from any Panc89 cell spheroids cultured with CD8^+^ T cells (**Figure 5D**).

Overall, these data demonstrate that Gemcitabine treatment diminishes the release of CD8^+^ T cell effector molecules and reduces cell surface expression level of PD-1 on CD8^+^ T cells. Since cell death induction was reduced in PancTu1 spheroids but enhanced in Panc89 spheroids cultured with CD8^+^ T cells after Gemcitabine treatment, these effects seem to be dependent on the PDAC cells.

## 4 Discussion

In many cancer entities, ICI treatment has emerged as a successful therapeutic option even at advanced disease stages prolonging the overall survival of the patients (21–23). However, PDAC patients have not shown significant treatment responses yet (24, 25). Still, the mechanisms of ICI resistance of PDAC are poorly understood, particularly regarding the metastatic setting. Besides expression of PD-L1 and PD-1 by tumor and stromal cells, a high mutational tumor burden and high infiltration and presence of CD8^+^ T cells are known prognostic parameters for the prediction of an ICI response (26–30). PDAC is characterized by a low mutational burden and regarded as a so called “cold” or “excluded” tumor with a general low T cell infiltration into the tumor (30). In addition, CD8^+^ T cells are often defined as exhausted and show a higher resistance towards ICI treatment (31, 32). Altogether, these findings provide an explanation for ICI resistance of this tumor (33). However, these parameters have been described for primary PDAC and still knowledge about expression of PD-L1 and stromal composition in PDAC metastases is rare and whether immune evasion of metastatic PDAC relies on the PD-L1/PD-1 axis.

Rahn et al. showed that PD-L1 is expressed in primary tumor tissues of PDAC patients staged T3N1M0, however, only in 20% of the analyzed tissues (9). As 80% of PDAC patients are diagnosed at a locally advanced or metastatic stage, with the liver representing the main site of metastases (2, 34), it was first to be determined whether PD-L1 is expressed in liver metastases of PDAC patients and how this relates to the abundance and spatial distribution of CD8^+^ T cells. In this study, IHC stainings of liver tissue sections revealed that during metastatic progression (from small to large metastases) the tumor microenvironment (TME) seems to be remodeled. Staining of αSMA, indicative for myofibroblasts, was less detectable in small metastases compared to large metastases which is in line with the findings of Quaranta et al., who showed in a PDAC mouse model that αSMA expression was lower in small metastatic lesions compared to large liver metastases (20). Myofibroblasts are the main source of ECM molecules such as collagen or fibronectin within the desmoplastic tumor stroma (11). The ECM is a physical barrier for T cells and has been shown to prevent the penetration of CD8^+^ T cells into the liver metastases (12). The inability of CD3^+^ and CD8^+^ T cells to infiltrate into metastases was also observed in our cohort, particularly in large metastases. Here, CD3^+^ T cells and CD8^+^ T cells, respectively were mostly found at the invasion front, while in small metastases CD8^+^ T cells were detected throughout the whole lesions or in the tumor center. Since the proportion of myofibroblasts was higher in large metastases, it can be speculated that myofibroblasts along with the released ECM molecules provide a physical barrier preventing T cell infiltration into metastases. Quaranta et al. demonstrated similar results in liver metastases of PDAC patients. Here, the number of CD8^+^ T cells was higher and closer to PanCK poor lesions (reflecting small lesions) compared to PanCK rich lesions (reflecting large lesions) (20). Grout et al. also showed that in non-small-cell lung cancer (NSCLC) tumor infiltration by CD3^+^ and CD8^+^ T cells was significantly lower when αSMA^+^ CAF are present at the invasion front (35). Finally, PD-L1 expression was mainly located at the invasion front or evenly distributed in large metastases, while small metastases either lacked PD-L1 expression or showed mostly weak expression in the center. Double staining revealed that PD-L1 is predominantly expressed by stromal cells, especially HMF. Besides HMF also Kupffer cells (36) or other metastases-associated macrophages seemed to be an important source of tumoral PD-L1 expression. The fact that in PDAC stromal cells appear to be the main source of PD-L1 expression rather than the PDAC cells has been already demonstrated by Rahn et al. in primary tumors of PDAC patients (9). Of note, small liver metastases with no or low PD-L1 expression comprised more CD8^+^ T cells in the tumor center, while large metastases exhibiting stronger PD-L1 expression comprised less CD8^+^ T cells being mostly located at the invasion front. Overall, these findings led to the conclusion that HMF associated PD-L1 expression might contribute to immune evasion of PDAC cells.

These differences between small and large metastases were experimentally well mimicked in 3D cocultures *in vitro* by using different tumor cell to HMF ratios (3:1 ratio = large and 5:1 ratio = small metastases). The fact that the diameter of the spheroids was larger after coculture with HMF along with a higher number of PDAC cells (data not shown) indicates that HMF support tumor cell growth, which is in line with previous findings from other *in vivo* and *in vitro* studies (14, 37). As observed in the liver metastases, HMF exhibited higher PD-L1 expression compared to PDAC cells. Moreover, PD-L1 cell surface levels on HMF were altered in the presence of PDAC cells, and HMF were able to increase PD-L1 expression on PDAC cell lines. In line with these findings, Inoue et al. reported that patients with lung adenocarcinoma classified with a high expression of αSMA were PD-L1 positive suggesting a correlation between a high abundance of myofibroblasts and elevated PD-L1 expression. Furthermore, CAF have been shown to increase PD-L1 expression on different lung adenocarcinoma cell lines *via* CXCL2 secretion and signaling (32). CXCL2 can activate the JAK-STAT signaling pathway, resulting in the upregulation of transcriptional factors that control the expression of PD-L1 (38). Therefore, strong PD-L1 expression and high local abundance of myofibroblasts might be also mechanistically linked in hepatic PDAC metastases. Gorchs et al. also showed that CAF isolated from tumor tissues of PDAC patients enhance the expression of immune checkpoint molecules, like PD-1 and CTLA-4, on CD8^+^ and CD4^+^ T cells (13). In PDAC tissues, most CD8^+^ T cells are exhausted and show a high expression of PD-1 (39). Likewise, CD8^+^ T cells used in our 3D cultures were characterized by high PD-1 expression. In a PDAC mouse model, it was shown that CD8^+^ T cells around large liver metastases showed higher PD-1 expression compared to those located around small metastases (20) further pointing to a role of the PD-L1/PD-1 axis in immune evasion of PDAC. Indeed, ccK18 levels were lower in supernatants of CD8^+^ T cells cultured with PDAC coculture spheroids, particularly when a high amount of HMF was present in the spheroids, indicating a reduced PDAC cell death induction the more HMF are abundant. This finding is in line with the lower cell surface expression levels of activation markers on CD8^+^ T cells as well as lower levels of cytotoxic T cell associated effector molecules in the supernatants, particularly of culture with Panc89 HMF spheroids.

In order to investigate whether the PD-1/PD-L1 inhibitory axis is involved in the HMF mediated impairment of CD8^+^ T cells in our stroma enriched PDAC spheroid models, treatment with the clinically validated PD-L1 and PD-1 inhibitors Durvalumab and Pembrolizumab, respectively, was performed. In line with results from recent clinical trials that tested the benefit of ICI for the treatment of PDAC patients (5, 31, 40), neither Durvalumab nor Pembrolizumab treatment improved the effector phenotype of CD8^+^ T cells and increased PDAC cell death in our human stroma enriched 3D PDAC model. In this context, it has to be critically noted that based on our current imaging modalities, no reliable conclusion can be made whether CD8^+^ T cells infiltrate differentially into HMF enriched spheroids compared to monoculture PDAC spheroids and whether this is impacted by ICI. For obtaining clear information regarding the spatial distribution of CD8^+^ T cells and the other cell components in our spheroids, ongoing studies have been started to fix the spheroids in formalin and embed them in paraffin for sectioning and immunohistochemical stainings as performed with the liver metastases. Nevertheless, overall these findings support the view that PD-1/PD-L1 axis may not be the major regulator of T cell mediated immunity in liver metastases of PDAC and rather indicate that other mechanisms are more relevant.

In this context, it has been shown that signaling *via* the tumor suppressor protein p53 can upregulate expression of CD80 on tumor cells, which in turn causes T cell suppression *via* CTLA-4 signaling (41). Further, Yazdanifar et al. showed that pancreatic cancer cells express Galectin-9, which can inhibit CD8^+^ T cells by binding to T cell immunoglobulin and mucin domain-containing protein 3 (TIM3) (42). The expression of different mucins, like MUC1, MUC4, and MUC16, which are upregulated during cancer progression, may also inhibit apoptosis of tumor cells (43). Other reported mechanisms that affect the resistance towards ICI might be: downregulation of the major histocompatibility complex I (MHCI), resulting in less antigen recognition, loss of IFNγ sensitivity due to IFNγ receptor mutations or deletions, an immunosuppressive TME or upregulation of other immune checkpoint regulators (TIM3, LAG3, CTLA-4) (44). Another mediator of ICI resistance might be a hypoxic microenvironment. PDAC is a hypoxic tumor (45) and in other cancer entities tumor hypoxia has been identified as a physical and molecular driver of resistance towards PD-1 blockade (32, 46). Furthermore, Thomas et al. and Trapani et al. showed that TGF-β secreted by myofibroblasts inhibits CD8^+^ T cells, especially the expression of genes encoding for cytotoxic effector molecules (47, 48). In our spheroid models, TGF-β1 levels in supernatants of PancTu1 coculture spheroids were slightly elevated compared to the respective monoculture, especially in coculture spheroids containing the highest proportion of HMF, fitting together with the reduced PDAC cell death under these conditions (**Supplementary Figure 5A**). However, in supernatants of Panc89 coculture spheroids TGF-β1 levels were not altered, suggesting that also other mechanisms contribute to the impairment of the T cell effector phenotype (**Supplementary Figure 5B**). Besides Granzyme A/B, Perforin, Granulysin, and IFNγ, CD8^+^ T cells secrete various other effector molecules by which they are able to induce cell death in their target cells. One of these mediators is FasL (CD95), which binds to its membrane-bound receptor Fas (CD95) and, thereby, initiates caspase-mediated apoptosis. In 2D cocultures of CD8^+^ T cells with HMF, we observed lower FasL levels in comparison to respective monocultured CD8^+^ T cells (data not shown), indicating that effector molecules like FasL might also play a role in our spheroid culture system. Rashid et al. showed that PDAC cell lines exhibit different cell surface levels of Fas, which can be altered by Gemcitabine (49).

Of note, pretreatment of Panc89 spheroids with Gemcitabine reversed the HMF reduced PDAC cell death in the presence of CD8^+^ T cells, although activation markers and release of cytotoxic molecules were decreased. Interestingly, the secretion of TGF-β1 was reduced in Panc89 spheroids pretreated with Gemcitabine compared to untreated spheroids, providing an explanation for the observed elevated PDAC cell death induction under these conditions (**Supplementary Figure 5D**). However, although Gemcitabine is one of the first-line therapies for PDAC patients, it only slightly prolongs the overall survival (50). One important reason for this is the often intrinsic or acquired resistance of PDAC cells which can be also seen in PDAC cell lines (51). Accordingly, PancTu1 cells have been described as chemoresistant, as apoptosis could not or only slightly be induced by Gemcitabine (52). This is in line with the findings of our study, in which treatment with Gemcitabine led to slightly lower ccK18 levels in supernatants from PancTu1 spheroids compared to Panc89 spheroids. Furthermore, sensitivity towards Gemcitabine treatment was reduced in both PancTu1 and Panc89 spheroids cocultured with HMF, underscoring the impact of myofibroblasts on the mediation of drug resistance in PDAC (53, 54). Of note, Gemcitabine treatment did not alter PD-L1 expression on PDAC cells and HMF but significantly diminished PD-1 surface levels on CD8^+^ T cells. Considering this finding, Gemcitabine treatment might also impair therapeutic efficiency of ICI treatment in PDAC patients

As outlined above, HMF show high expression of PD-L1 and clearly exert immunosuppressive effects on CD8^+^ T cells, but this seemed to be independent of or at least not exclusively dependent on the PD-L1/PD-1 axis. Therefore, combinational therapeutic strategies simultaneously targeting different immune checkpoints or different tumor promoting stromal cells, might be a more effective approach to overcome immunosuppression leading to tumor elimination of PDAC, as recently demonstrated in PDAC mouse models (55, 56).

## Data availability statement

The original contributions presented in the study are included in the article/**Supplementary Material**. Further inquiries can be directed to the corresponding author.

## Ethics statement

The studies involving human participants were reviewed and approved by the ethics committee of the Medical Faculty of Kiel University (reference number: A110/99 and D490/17). The patients/participants provided their written informed consent to participate in this study.

## Author contributions

Conceptualization: SB, TD, and SS. Methodology: SB, TD, LA, SR, SK, and SH. Investigation: SB, TD, and LA. Resources: SS, SR, HS, DW, and CR. Visualization: SB. Funding acquisition: SS. Writing—original draft preparation: SB, TD, and SS. Writing—review and editing: all authors. All authors contributed to the article and approved the submitted version.

## Supplementary material

Supplementary Figure 1 Analysis of small and large PDAC metastases in liver tissue sections.**(A)** Representative image of PanCK stained liver tissue sections obtained from a PDAC patient showing small and large metastatic lesions. **(B)** Number of small and large metastases in all analyzed liver sections of PDAC patients. Proportion and localization (discriminated into tumor center and invasion front) of **(C)** CD8^+^ and **(D)** PD-L1^+^ cells as well as **(E)** intensity of PD-L1 staining in small and large metastases. Data represents the mean ± SD of 15 independent liver tissue sections. * = p < 0.05, ** = p < 0.01, *** p < 0.001.

Supplementary Figure 2 αSMA^+^ myofibroblasts and CD68^+^ macrophages are the main PD-L1 expressing cells in liver metastases of PDAC patients. Representative images of double immunohistochemical staining of **(A)** PanCK/PD-L1, **(B)** αSMA/PD-L1, and **(C)** CD68/PD-L1 in a large liver metastasis of a PDAC patient at 200 x (left images) and 400 x magnification (right images). Arrows indicate PD-L1 staining in αSMA^+^ myofibroblasts or CD68^+^ macrophages.

Supplementary Figure 3 Durvalumab binds to PD-L1 on PDAC cells and hepatic myofibroblasts. PancTu1 and Panc89 cells were either seeded in mono- or in coculture with hepatic myofibroblasts (HMF) at different ratios in ultra-low attachment plates. After 48 h, either 10 μg/mlisotype control or Durvalumab were added to spheroid cultures for 24 h. Afterwards **(A)** PancTu1 cells, **(B)** Panc89 cells, and **(C)** HMF were stained for cell surface localized PD-L1 and analyzed via flow cytometry. Data were normalized on the respective isotype control. MFI ratio for specific PD-L1 staining was determined. Data represents the mean ± SD (normally distributed). N=3. * = p < 0.05, ** = p < 0.01, *** = p < 0.001

Supplementary Figure 4 PD-L1 expression on PancTu1, Panc89 cells, and hepatic myofibroblasts after Gemcitabine treatment. PancTu1, Panc89 cells, and hepatic myofibroblasts(HMF) were seeded in monoculture in ultra-low attachment plates for 24 h. Then, cells were either left untreated or treated with 10 μg/ml Gemcitabine. After 24 h, spheroids were dissociated and stained for PD-L1 by flow cytometry analysis. MFI ratio of PD-L1 cell surface expression on PancTu1 cells, Panc89 cells, and HMF. Data represents the mean ± SD (normally distributed). N=3.

Supplementary Figure 5 TGF-β1 concentration in supernatants of PDAC mono- and coculture spheroids. PancTu1 and Panc89 cells were either seeded in mono- or coculture with hepatic myofibroblasts (HMF) at different ratios in ultra-low attachment plates. Concentration of TGF-β1 was measured in the supernatants of **(A)** PancTu1 spheroids and **(B)** Panc89 spheroids after 72 h of mono- or coculture. Data were normalized on the coculture with monocultured spheroids. Concentration of TGF-β1 was measured after a total duration of 72 h insupernatants of mono- or coculture **(C)** PancTu1 spheroids and **(D)** Panc89 spheroids, stimulated with 10 μg/ml Gemcitabine for 24 h. Data were normalized on the coculture with untreated spheroids. Data represents the mean ± SD (normally distributed). N=4. *** = p < 0.001

## References

[1] Siegel RL Miller KD Fuchs HE Jemal A. Cancer statistics, 2021. CA Cancer J Clin (2021) 71(1):7–33. doi: 10.3322/CAAC.21654 33433946

[2] Houg DS Bijlsma MF. The hepatic pre-metastatic niche in pancreatic ductal adenocarcinoma. Mol Cancer BioMed Cent Ltd (2018) 17:1–18. doi: 10.1186/s12943-018-0842-9 PMC6003100 29903049

[3] Guillén-Ponce C Blázquez J González I de-Madaria E Montáns J Carrato A. Diagnosis and staging of pancreatic ductal adenocarcinoma. Clin Transl Oncol (2017) 19(10):1205–16. doi: 10.1007/S12094-017-1681-7 28612200

[4] Ghaneh P Costello E Neoptolemos JP. Biology and management of pancreatic cancer. Postgrad Med J (2008) 84(995):478–97. doi: 10.1136/GUT.2006.103333 18940950

[5] Brahmer JR Tykodi SS Chow LQM Hwu WJ Topalian SL Hwu P. Safety and activity of anti–PD-L1 antibody in patients with advanced cancer. N Engl J Med (2012) 366(26):2455. doi: 10.1056/NEJMOA1200694 22658128 PMC3563263

[6] Patnaik A Kang SP Rasco D Papadopoulos KP Elassaiss-Schaap J Beeram M. Phase I study of pembrolizumab (MK-3475; anti-PD-1 monoclonal antibody) in patients with advanced solid tumors. Clin Cancer Res (2015) 21(19):4286–93. doi: 10.1158/1078-0432.CCR-14-2607 25977344

[7] Carstens JL De Sampaio PC Yang D Barua S Wang H Rao A. Spatial computation of intratumoral T cells correlates with survival of patients with pancreatic cancer. Nat Commun (2017) 8. doi: 10.1038/NCOMMS15095 PMC5414182 28447602

[8] Danilova L Ho WJ Zhu Q Vithayathil T De Jesus-Acosta A Azad NS. Programmed cell death ligand-1 (PD-L1) and CD8 expression profiling identify an immunologic subtype of pancreatic ductal adenocarcinomas with favorable survival. Cancer Immunol Res (2019) 7(6):886. doi: 10.1158/2326-6066.CIR-18-0822 31043417 PMC6548624

[9] Rahn S Krüger S Mennrich R Goebel L Wesch D Oberg HH. POLE score: a comprehensive profiling of programmed death 1 ligand 1 expression in pancreatic ductal adenocarcinoma. Oncotarget (2019) 10(16):1572–88. doi: 10.18632/oncotarget.26705 PMC6422186 30899426

[10] Rasheed ZA Matsui W Maitra A. Pathology of pancreatic stroma in PDAC. In: Pancreat cancer tumor microenviron. Trivandrum (India): Transworld Research Network (2012). 22876385

[11] Pickup MW Mouw JK Weaver VM. The extracellular matrix modulates the hallmarks of cancer. EMBO Rep (2014) 15(12):1243–53. doi: 10.15252/EMBR.201439246 PMC4264927 25381661

[12] Aldag L Beckinger S Daunke T Philipp LM Surrow A Yesilyurt UU. The heterogeneity of the tumor microenvironment as essential determinant of development, progression and therapy response of pancreatic cancer. Cancers (Basel) (2021) 13(19):4932. doi: 10.3390/CANCERS13194932 34638420 PMC8508450

[13] Gorchs L Moro CF Bankhead P Kern KP Sadeak I Meng Q. Human pancreatic carcinoma-associated fibroblasts promote expression of co-inhibitory markers on CD4+ and CD8+ T-cells. Front Immunol (2019) 10:847(APR). doi: 10.3389/fimmu.2019.00847 31068935 PMC6491453

[14] Lenk L Pein M Will O Gomez B Viol F Hauser C. The hepatic microenvironment essentially determines tumor cell dormancy and metastatic outgrowth of pancreatic ductal adenocarcinoma. Oncoimmunology (2018) 7(1). doi: 10.1080/2162402X.2017.1368603 PMC5739558 29296518

[15] Skorupan N Palestino Dominguez M Ricci SL Alewine C. Clinical strategies targeting the tumor microenvironment of pancreatic ductal adenocarcinoma. Cancers (Basel) (2022) 14(17). doi: 10.3390/CANCERS14174209 PMC9454553 36077755

[16] Carvalho TMA Di Molfetta D Greco MR Koltai T Alfarouk KO Reshkin SJ. Tumor microenvironment features and chemoresistance in pancreatic ductal adenocarcinoma: insights into targeting physicochemical barriers and metabolism as therapeutic approaches. Cancers (Basel) (2021) 13(23):6135. doi: 10.3390/CANCERS13236135 34885243 PMC8657427

[17] Shields MA Dangi-Garimella S Redig AJ Munshi HG. Biochemical role of the collagen-rich tumour microenvironment in pancreatic cancer progression. Biochem J (2012) 441(2):541. doi: 10.1042/BJ20111240 22187935 PMC8215985

[18] Moore PS Sipos B Orlandini S Sorio C Real FX Lemoine NR. Genetic profile of 22 pancreatic carcinoma cell lines. Anal K-ras p53 p16 DPC4/Smad4 Virchows Arch (2001) 439(6):798–802. doi: 10.1007/S004280100474 11787853

[19] Sipos B Möser S Kalthoff H Török V Löhr M Klöppel G. A comprehensive characterization of pancreatic ductal carcinoma cell lines: towards the establishment of an *in vitro* research platform. Virchows Arch (2003) 442(5):444–52. doi: 10.1007/S00428-003-0784-4 12692724

[20] Quaranta V Rainer C Nielsen SR Raymant ML Ahmed MS Engle DD. Macrophage-derived granulin drives resistance to immune checkpoint inhibition in metastatic pancreatic cancer. Cancer Res (2018) 78(15):4253–69. doi: 10.1158/0008-5472.CAN-17-3876 PMC6076440 29789416

[21] Xu L Yan X Ding W. Meta-analysis of efficacy from CTLA-4 and PD-1/PD-L1 inhibitors in cancer patients. Front Oncol (2022) 12:1460. doi: 10.3389/fonc.2022.876098 PMC9097585 35574317

[22] Xu Y Wang Q Xie J Chen M Liu H Zhan P. The predictive value of clinical and molecular characteristics or immunotherapy in non-small cell lung cancer: a meta-analysis of randomized controlled trials. Front Oncol (2021) :11:3605. doi: 10.3389/fonc.2021.732214 PMC8453160 34557415

[23] Zou Y Zou X Zheng S Tang H Zhang L Liu P. Efficacy and predictive factors of immune checkpoint inhibitors in metastatic breast cancer: a systematic review and meta-analysis. Ther Adv Med Oncol (2020) 12. doi: 10.1177/1758835920940928 PMC7436841 32874208

[24] Kabacaoglu D Ciecielski KJ Ruess DA Algül H. Immune checkpoint inhibition for pancreatic ductal adenocarcinoma: current limitations and future options. Front Immunol (2018) 9:1878(AUG). doi: 10.3389/FIMMU.2018.01878 30158932 PMC6104627

[25] Wandmacher AM Letsch A Sebens S. Challenges and future perspectives of immunotherapy in pancreatic cancer. Cancers (Basel) (2021) 13(16). doi: 10.3390/CANCERS13164235 PMC8391691 34439389

[26] Patel SP Kurzrock R. PD-L1 expression as a predictive biomarker in cancer immunotherapy. Mol Cancer Ther (2015) 14(4):847–56. doi: 10.1158/1535-7163.MCT-14-0983/86482/AM/PD-L1-EXPRESSION-AS-A-PREDICTIVE-BIOMARKER-IN 25695955

[27] Noguchi T Ward JP Gubin MM Arthur CD Lee SH Hundal J. Temporally distinct PD-L1 expression by tumor and host cells contributes to immune escape. Cancer Immunol Res (2017) 5(2):106–17. doi: 10.1158/2326-6066.CIR-16-0391 PMC5510474 28073774

[28] Lau J Cheung J Navarro A Lianoglou S Haley B Totpal K. Tumour and host cell PD-L1 is required to mediate suppression of anti-tumour immunity in mice. Nat Commun (2017) 8. doi: 10.1038/NCOMMS14572 PMC5321797 28220772

[29] Strickler JH Hanks BA Khasraw M. Tumor mutational burden as a predictor of immunotherapy response: is more always better? Clin Cancer Res (2021) 27(5):1236–41. doi: 10.1158/1078-0432.CCR-20-3054 PMC9912042 33199494

[30] Zhou X Ni Y Liang X Lin Y An B He X. Mechanisms of tumor resistance to immune checkpoint blockade and combination strategies to overcome resistance. Front Immunol (2022) 13:915094. doi: 10.3389/FIMMU.2022.915094 36189283 PMC9520263

[31] Mukherji R Debnath D Hartley ML Noel MS. The role of immunotherapy in pancreatic cancer. Curr Oncol (2022) 29(10):6864. doi: 10.3390/CURRONCOL29100541 36290818 PMC9600738

[32] Zandberg DP Menk AV Velez M Normolle D Depeaux K Liu A. Tumor hypoxia is associated with resistance to PD-1 blockade in squamous cell carcinoma of the head and neck. J Immunother Cancer (2021) 9(5). doi: 10.1136/JITC-2020-002088 PMC8126285 33986123

[33] Lawlor RT Mattiolo P Mafficini A Hong SM Piredda ML Taormina SV. Tumor mutational burden as a potential biomarker for immunotherapy in pancreatic cancer: systematic review and still-open questions. Cancers (Basel) (2021) 13(13):3119. doi: 10.3390/CANCERS13133119/S1 34206554 PMC8269341

[34] Yachida S White CM Naito Y Zhong Y Brosnan JA Macgregor-Das AM. Clinical significance of the genetic landscape of pancreatic cancer and implications for identification of potential long-term survivors. Clin Cancer Res (2012) 18(22):6339–47. doi: 10.1158/1078-0432.CCR-12-1215 PMC3500447 22991414

[35] Grout JA Sirven P Leader AM Maskey S Hector E Puisieux I. Spatial positioning and matrix programs of cancer-associated fibroblasts promote T-cell exclusion in human lung tumors. Cancer Discovery (2022) 12(11):2606–25. doi: 10.1158/2159-8290.CD-21-1714 PMC9633420 36027053

[36] Iwai Y Terawaki S Ikegawa M Okazaki T Honjo T. PD-1 inhibits antiviral immunity at the effector phase in the liver. J Exp Med (2003) 198(1):39. doi: 10.1084/JEM.20022235 12847136 PMC2196084

[37] Sun Q Zhang B Hu Q Qin Y Xu W Liu W. The impact of cancer-associated fibroblasts on major hallmarks of pancreatic cancer. Theranostics (2018) 8(18):5072. doi: 10.7150/THNO.26546 30429887 PMC6217060

[38] Lee SJ Jang BC Lee SW Yang Y Suh S II Park YM. Interferon regulatory factor-1 is prerequisite to the constitutive expression and IFN-gamma-induced upregulation of B7-H1 (CD274). FEBS Lett (2006) 580(3):755–62. doi: 10.1016/J.FEBSLET.2005.12.093 16413538

[39] Saka D Gökalp M Piyade B Cevik NC Sever EA Unutmaz D. Mechanisms of t-cell exhaustion in pancreatic cancer. Cancers MDPI AG; (2020) 12:1–27. doi: 10.3390/cancers12082274 PMC7464444 32823814

[40] Royal RE Levy C Turner K Mathur A Hughes M Kammula US. Phase 2 trial of single agent ipilimumab (Anti-CTLA-4) for locally advanced or metastatic pancreatic adenocarcinoma. J Immunother (2010) 33(8):828–33. doi: 10.1097/CJI.0B013E3181EEC14C PMC7322622 20842054

[41] Scarpa M Marchiori C Scarpa M Castagliuolo I. CD80 expression is upregulated by TP53 activation in human cancer epithelial cells. Oncoimmunology (2021) 10(1). doi: 10.1080/2162402X.2021.1907912 PMC8023236 33868791

[42] Yazdanifar M Zhou R Grover P Williams C Bose M Moore LJ. Overcoming immunological resistance enhances the efficacy of a novel anti-tMUC1-CAR T cell treatment against pancreatic ductal adenocarcinoma. Cells (2019) 8(9). doi: 10.3390/CELLS8091070 PMC6770201 31514488

[43] Suh H Pillai K Morris DL. Mucins in pancreatic cancer: biological role, implications in carcinogenesis and applications in diagnosis and therapy. Am J Cancer Res (2017) 7(6):1372. 28670497 PMC5489784

[44] Schoenfeld AJ Hellmann MD. Acquired resistance to immune checkpoint inhibitors. Cancer Cell (2020) 37(4):443. doi: 10.1016/J.CCELL.2020.03.017 32289269 PMC7182070

[45] Koong AC Mehta VK Le QT Fisher GA Terris DJ Brown JM. Pancreatic tumors show high levels of hypoxia. Int J Radiat Oncol Biol Phys (2000) 48(4):919–22. doi: 10.1016/S0360-3016(00)00803-8 11072146

[46] Najjar YG Menk AV Sander C Rao U Karunamurthy A Bhatia R. Tumor cell oxidative metabolism as a barrier to PD-1 blockade immunotherapy in melanoma. JCI Insight (2019) 4(5). doi: 10.1172/JCI.INSIGHT.124989 PMC6483505 30721155

[47] Trapani JA. The dual adverse effects of TGF-beta secretion on tumor progression. Cancer Cell (2005) 8(5):349–50. doi: 10.1016/J.CCR.2005.10.018 16286241

[48] Thomas DA Massagué J. TGF-beta directly targets cytotoxic T cell functions during tumor evasion of immune surveillance. Cancer Cell (2005) 8(5):369–80. doi: 10.1016/J.CCR.2005.10.012 16286245

[49] Rashid K Röder C Goumas F Egberts JH Kalthoff H. CD95L inhibition impacts gemcitabine-mediated effects and non-apoptotic signaling of tnf-α and trail in pancreatic tumor cells. Cancers (Basel) (2021) 13(21):5458. doi: 10.3390/CANCERS13215458/S1 34771621 PMC8582466

[50] Burris HA Moore MJ Andersen J Green MR Rothenberg ML Modiano MR. Improvements in survival and clinical benefit with gemcitabine as first-line therapy for patients with advanced pancreas cancer: a randomized trial. J Clin Oncol (1997) 15(6):2403–13. doi: 10.1200/JCO.1997.15.6.2403 9196156

[51] Liu K Geng Y Wang L Xu H Zou M Li Y. Systematic exploration of the underlying mechanism of gemcitabine resistance in pancreatic adenocarcinoma. Mol Oncol (2022) 16(16):3034–51. doi: 10.1002/1878-0261.13279 PMC9394232 35810469

[52] Arlt A Gehrz A Müerköster S Vorndamm J Kruse ML Fölsch UR. Role of NF-κB and Akt/PI3K in the resistance of pancreatic carcinoma cell lines against gemcitabine-induced cell death. Oncogene (2003) 22(21):3243–51. doi: 10.1038/sj.onc.1206390 12761494

[53] Müerköster S Wegehenkel K Arlt A Witt M Sipos B Kruse ML. Tumor stroma interactions induce chemoresistance in pancreatic ductal carcinoma cells involving increased secretion and paracrine effects of nitric oxide and interleukin-1β. Cancer Res (2004) 64(4):1331–7. doi: 10.1002/1878-0261.13279 14973050

[54] Neumann CCM von Hörschelmann E Reutzel-Selke A Seidel E Sauer IM Pratschke J. Tumor–stromal cross-talk modulating the therapeutic response in pancreatic cancer. Hepatobiliary Pancreat Dis Int (2018) 17(5):461–72. doi: 10.1016/j.hbpd.2018.09.004 30243879

[55] Kim DK Jeong J Lee DS Hyeon DY Park GW Jeon S. PD-L1-directed PlGF/VEGF blockade synergizes with chemotherapy by targeting CD141+ cancer-associated fibroblasts in pancreatic cancer. Nat Commun (2022) 13(1):1–19. doi: 10.1038/s41467-022-33991-6 36272973 PMC9588060

[56] Gulhati P Schalck A Jiang S Shang X Wu CJ Hou P. Targeting T cell checkpoints 41BB and LAG3 and myeloid cell CXCR1/CXCR2 results in antitumor immunity and durable response in pancreatic cancer. Nat Cancer (2022) 2022:1–19. doi: 10.1038/s43018-022-00500-z PMC9925045 36585453

