# Supplementary figures and images for: Hepatic myofibroblasts exert immunosuppressive effects independent of the immune checkpoint regulator PD-L1 in liver metastasis of pancreatic ductal adenocarcinoma
Source: Front Oncol. 2023 May 3;13:1160824. doi: 10.3389/fonc.2023.1160824 (PMC10189124; doi:10.3389/fonc.2023.1160824)

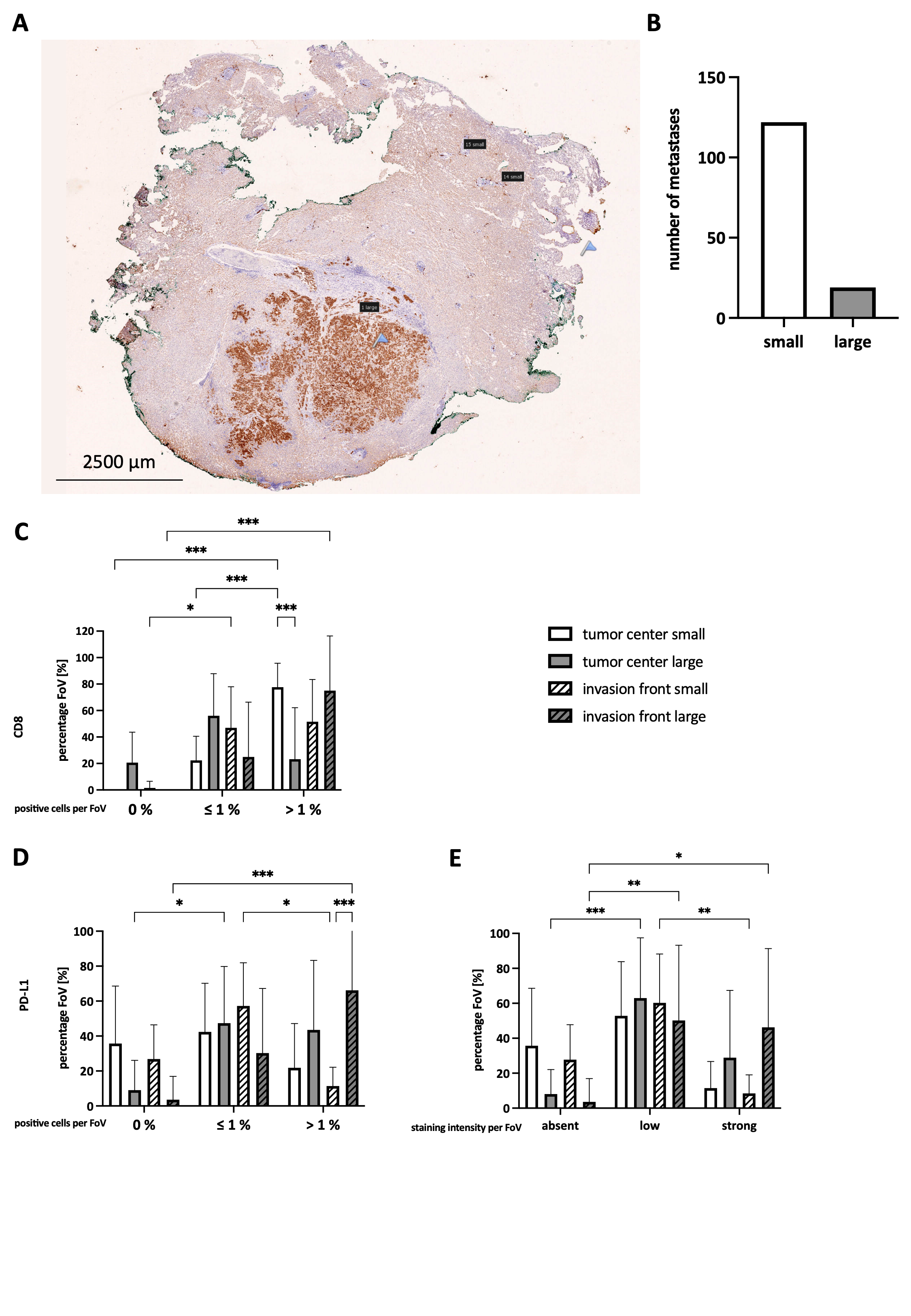

Supplement: Supplementary Figure 1 — Analysis of small and large PDAC metastases in liver tissue sections.(A) Representative image of PanCK stained liver tissue sections obtained from a PDAC patient showing small and large metastatic lesions. (B) Number of small and large metastases in all analyzed liver sections of PDAC patients. Proportion and localization (discriminated into tumor center and invasion front) of (C) CD8+ and (D) PD-L1+ cells as well as (E) intensity of PD-L1 staining in small and large metastases. Data represents the mean ± SD of 15 independent liver tissue sections. * = p < 0.05, ** = p < 0.01, *** p < 0.001. [file Image_1.tiff]

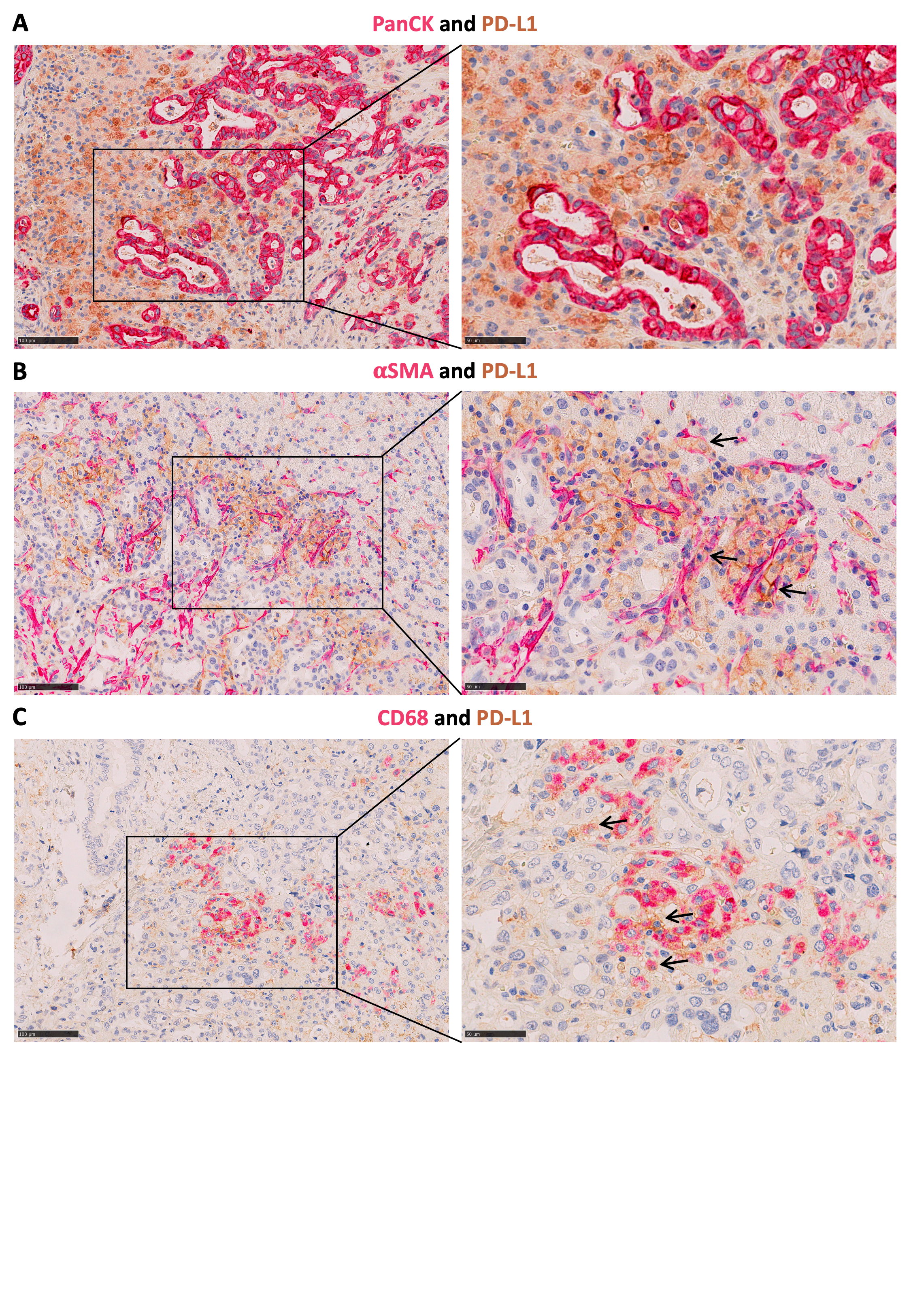

Supplement: Supplementary Figure 2 — αSMA+ myofibroblasts and CD68+ macrophages are the main PD-L1 expressing cells in liver metastases of PDAC patients. Representative images of double immunohistochemical staining of (A) PanCK/PD-L1, (B) αSMA/PD-L1, and (C) CD68/PD-L1 in a large liver metastasis of a PDAC patient at 200 x (left images) and 400 x magnification (right images). Arrows indicate PD-L1 staining in αSMA+ myofibroblasts or CD68+ macrophages. [file Image_2.tiff]

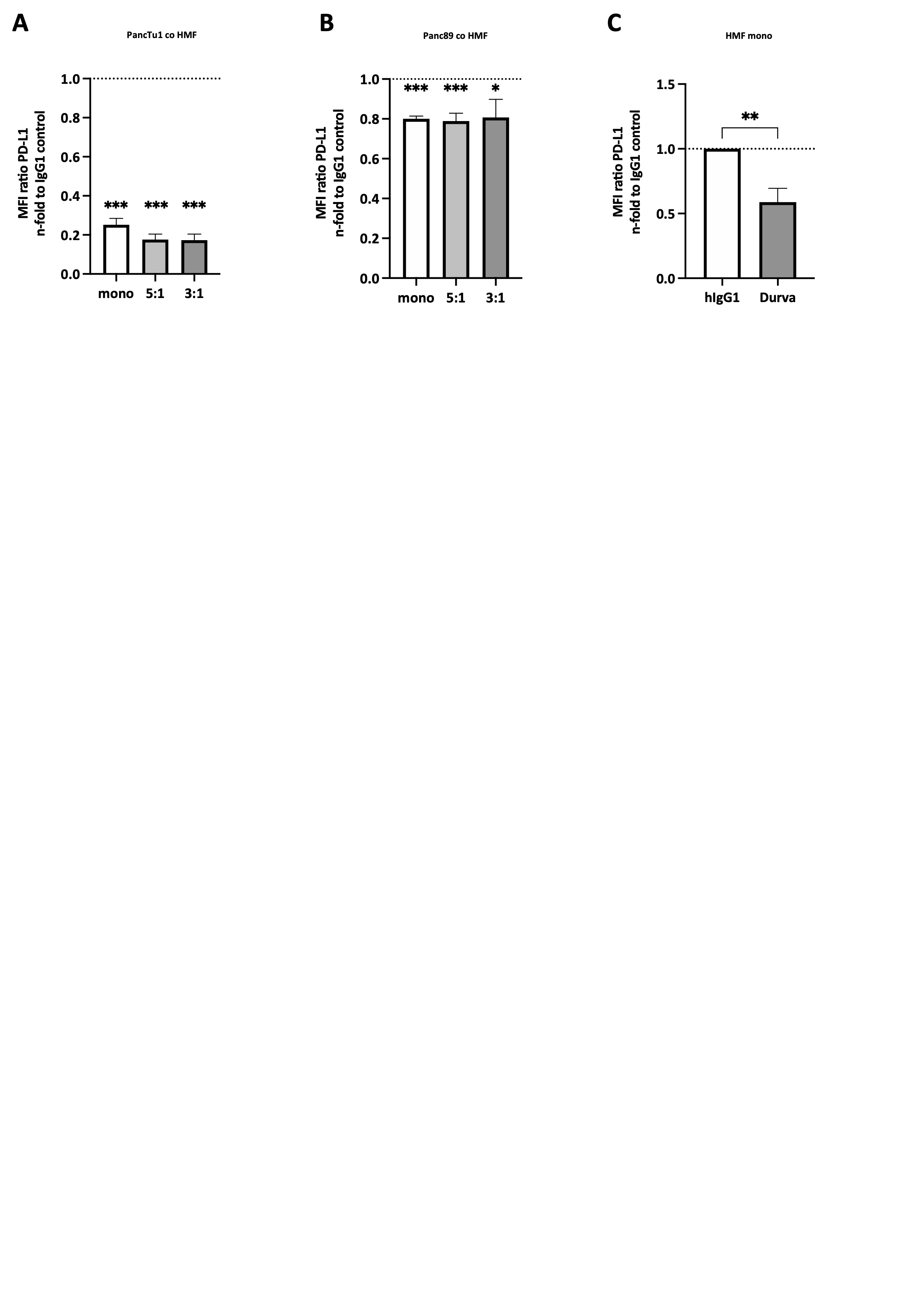

Supplement: Supplementary Figure 3 — Durvalumab binds to PD-L1 on PDAC cells and hepatic myofibroblasts. PancTu1 and Panc89 cells were either seeded in mono- or in coculture with hepatic myofibroblasts (HMF) at different ratios in ultra-low attachment plates. After 48 h, either 10 μg/mlisotype control or Durvalumab were added to spheroid cultures for 24 h. Afterwards (A) PancTu1 cells, (B) Panc89 cells, and (C) HMF were stained for cell surface localized PD-L1 and analyzed via flow cytometry. Data were normalized on the respective isotype control. MFI ratio for specific PD-L1 staining was determined. Data represents the mean ± SD (normally distributed). N=3. * = p < 0.05, ** = p < 0.01, *** = p < 0.001 [file Image_3.tiff]

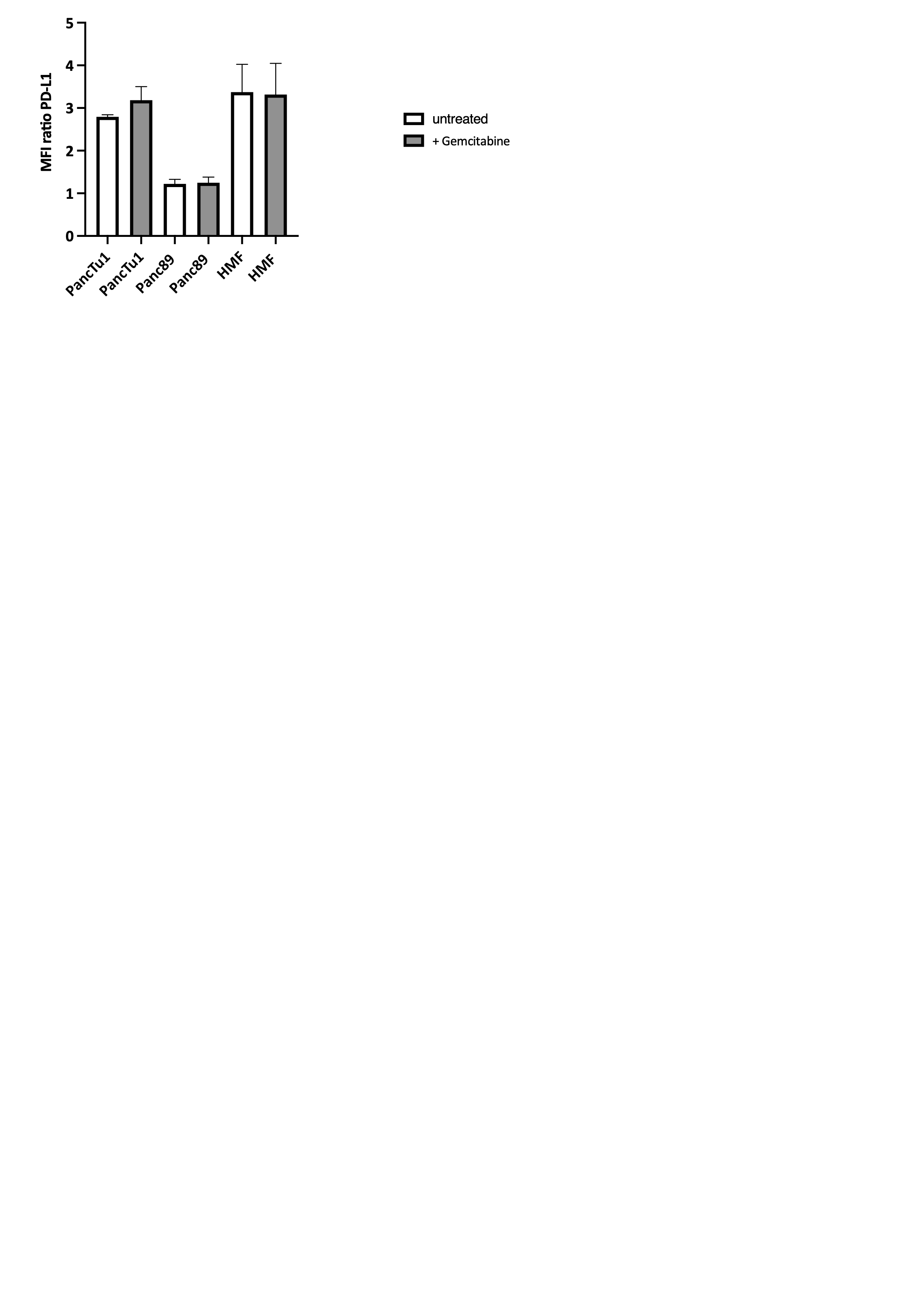

Supplement: Supplementary Figure 4 — PD-L1 expression on PancTu1, Panc89 cells, and hepatic myofibroblasts after Gemcitabine treatment. PancTu1, Panc89 cells, and hepatic myofibroblasts(HMF) were seeded in monoculture in ultra-low attachment plates for 24 h. Then, cells were either left untreated or treated with 10 μg/ml Gemcitabine. After 24 h, spheroids were dissociated and stained for PD-L1 by flow cytometry analysis. MFI ratio of PD-L1 cell surface expression on PancTu1 cells, Panc89 cells, and HMF. Data represents the mean ± SD (normally distributed). N=3. [file Image_4.tiff]

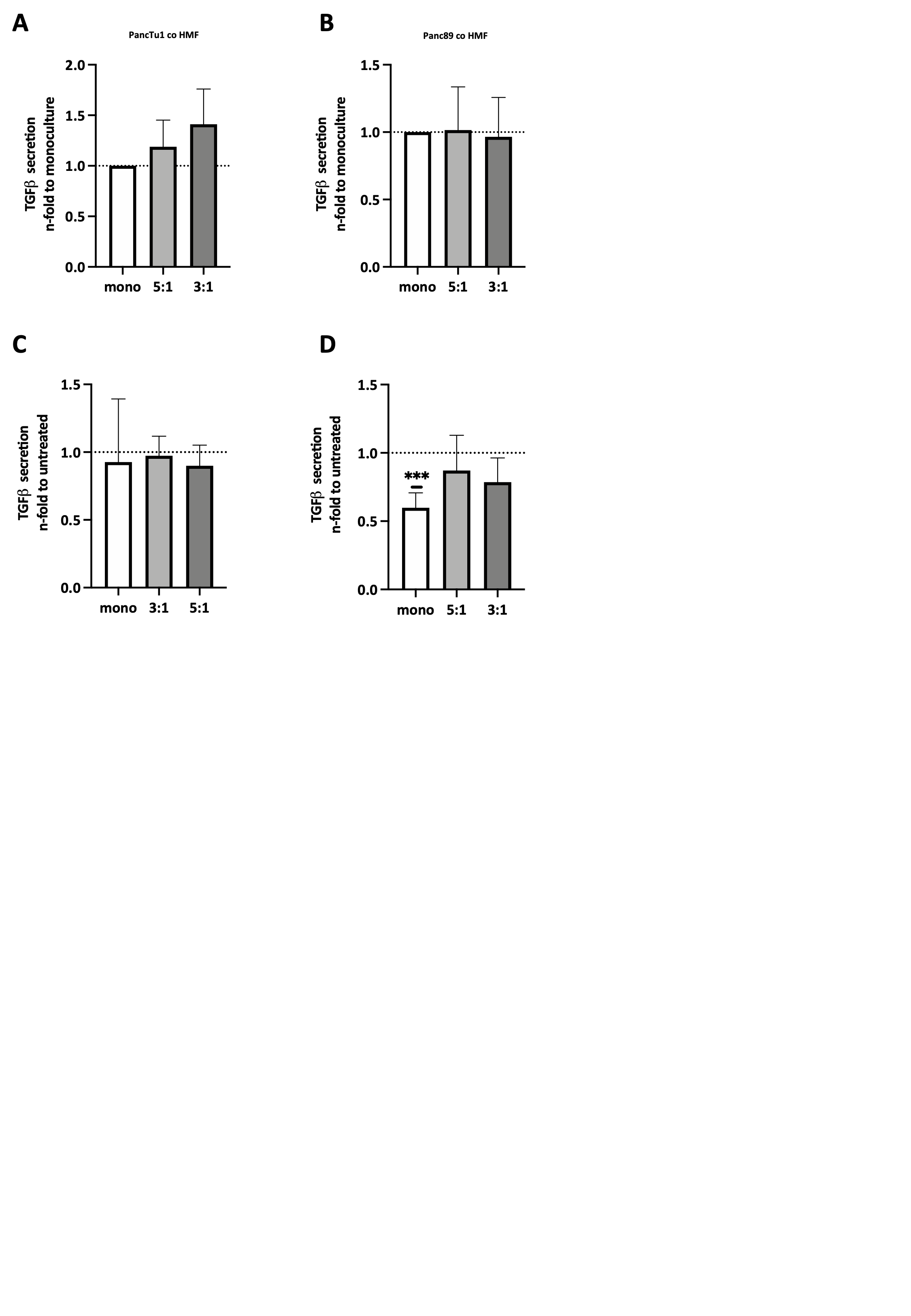

Supplement: Supplementary Figure 5 — TGF-β1 concentration in supernatants of PDAC mono- and coculture spheroids. PancTu1 and Panc89 cells were either seeded in mono- or coculture with hepatic myofibroblasts (HMF) at different ratios in ultra-low attachment plates. Concentration of TGF-β1 was measured in the supernatants of (A) PancTu1 spheroids and (B) Panc89 spheroids after 72 h of mono- or coculture. Data were normalized on the coculture with monocultured spheroids. Concentration of TGF-β1 was measured after a total duration of 72 h insupernatants of mono- or coculture (C) PancTu1 spheroids and (D) Panc89 spheroids, stimulated with 10 μg/ml Gemcitabine for 24 h. Data were normalized on the coculture with untreated spheroids. Data represents the mean ± SD (normally distributed). N=4. *** = p < 0.001 [file Image_5.tiff]
